# Towards more realistic measures of accessibility to emergency departments in Sweden

**DOI:** 10.1186/s12942-024-00364-9

**Published:** 2024-03-02

**Authors:** Jacob Hassler, Tobias Andersson Granberg, Krisjanis Steins, Vania Ceccato

**Affiliations:** 1 Department of Urban Planning and Environment, KTH Royal Institute of Technology, Teknikringen 10 A, 10044 Stockholm, Sweden; 2 Department of Science and Technology, Linköping University/ITN, 60174 Norrköping, Sweden

**Keywords:** Accessibility, Emergency health care, Dynamic population data, Spatiotemporal analysis

## Abstract

**Background:**

Assuring that emergency health care (EHC) is accessible is a key objective for health care planners. Conventional accessibility analysis commonly relies on resident population data. However, the allocation of resources based on stationary population data may lead to erroneous assumptions of population accessibility to EHC.

**Method:**

Therefore, in this paper, we calculate population accessibility to emergency departments in Sweden with a geographical information system based network analysis. Utilizing static population data and dynamic population data, we investigate spatiotemporal patterns of how static population data over- or underestimates population sizes derived from temporally dynamic population data.

**Results:**

Our findings show that conventional measures of population accessibility tend to underestimate population sizes particularly in rural areas and in smaller ED’s catchment areas compared to urban, larger ED’s—especially during vacation time in the summer.

**Conclusions:**

Planning based on static population data may thus lead to inequitable distributions of resources. This study is motivated in light of the ongoing centralization of ED’s in Sweden, which largely depends on population sizes in ED’s catchment areas.

**Supplementary Information:**

The online version contains supplementary material available at 10.1186/s12942-024-00364-9.

## Background

Accessible health care is a fundamental part of a well-functioning society. In the most time-sensitive conditions, such as stroke, acute heart conditions and severe trauma, minutes until receiving medical intervention can be the difference between survival and death. Longer travel distances to the emergency department (ED) and time delays in reaching the ED have been associated with increasing risk of mortality [1, 2]. To assure as quick medical intervention as possible for patients, planners and decision makers generally aim at increasing the share of the population that can reach (or be reached by) health care resources within stipulated time thresholds in order to maximize population accessibility.

Hitherto, analysis of the populations’ accessibility to emergency health care (EHC) has generally relied on census data where the share of the population that can reach an ED within a certain time frame is calculated in geographical information systems (GIS). However, this type of analysis is flawed as the population is implicitly assumed to be stationary. In reality, individuals relocate in space over the course of the day when they travel to work, and spend leisure time in recreational areas at certain times of the day and in certain seasons of the year. Implicitly assuming stationarity of populations may thus lead to over- or underestimation of population accessibility in certain areas, at certain times of the day [3]. Without considering temporality there is a risk that planners "may end up making decisions based on unrealistic or false information regarding e.g.[…] social equity in terms of service provision.” [4].

Despite accessibility being an essentially dynamic concept [5], the mapping of populations is still largely constrained by the limitations of census data [6]. As others have noted, novel data sources such as location data from the mobile network should be “critically employed […] to address long-standing questions of social justice [and] inequality…” [7]. In this vein, we investigate how temporality can be included in the analysis of spatiotemporal patterns of population accessibility to ED’s in Sweden. The main novelty is that we compare accessibility measures derived from static population data (census) and dynamic population data (location data from the mobile network) to illuminate how conventional accessibility analyses may over- and underestimate shares of the total population in certain places, at certain times. This is important because if static population data is inaccurately reflecting the actual population size, resources may unknowingly be allocated in sub-optimal ways. We focus particularly on urban and rural differences. This is motivated by an ongoing large-scale centralization of ED’s in recent decades, both in Sweden [8], and internationally [2, 9–11] that tend to disfavor rural areas [12]. Closure of rural ED’s have also been argued to have “substantial consequences on patient outcomes, particularly among communities with limited resources for time-sensitive illnesses” [13].

### Aim and research questions

The aim of this study is to assess temporal variations in population accessibility to ED’s in Sweden and to compare accessibility measures based on static population data with measures based on dynamic population data. This is achieved by calculating travel times to the closest ED for different areas covering all of Sweden, and then estimating the share of the population that have access to an ED within various time thresholds (10, 20, 30, 60 min or more). Then, we compare resident population data (static) and location data from the mobile network (dynamic) to assess spatiotemporal clusters of under- or overestimation of population accessibility when using static population data as a base for analysis. Lastly, we show the impact that such over-and underestimations may have for planning by illustrating how ‘inaccurate’ resident population data is reflecting shares of the total population at different times of the year, using the catchment areas of Sweden’s ED’s as units of analysis.

The research questions were thus;

- What are the spatiotemporal patterns of population accessibility to ED’s in Sweden?
- Does population accessibility to ED’s based on static population data over- or underestimate compared to population accessibility estimated from location data from the mobile network, and are there variations in over- and underestimation over the day; between weekdays and weekends; and/or between months?
- Are there spatiotemporal clusters of over- and underestimation of population shares in Sweden’s ED’s catchment areas, and does over- and underestimation vary between different ED types?

The article is structured as follows; first, we define accessibility and how it is conceptualized in this study. This is followed by a definition of accessibility in the context of EHC planning, and a brief summary of research in this field and how mobile phone data has been utilized in research on accessibility. Then, the study area (Sweden) is presented. This is followed by a description of the data and methods used. The results are then presented and discussed, before the article is concluded with some finishing conclusions and reflections.

### Previous research

#### Defining accessibility and incorporating temporality

Conceptually, accessibility is difficult to define due to the complexity of determining the factors that affect it. Outlining several dimensions of accessibility, referring to economical, geographical and social aspects of the concept, Penchansky and Thomas [14] define access as a representation of “the degree of “fit” between the clients and the system”. Similarly, Levesque et al. [15] define access to health care as “the opportunity to reach and obtain appropriate health care services in situations of perceived need of care”. Accessibility is in other words determined by an interplay between supply-side factors related to the organization of health care resources, and demand-side factors related to the population. Accessibility has also been conceptualized as depending on spatial and aspatial dimensions. Factors such as health care financing and cultural understandings belong to the latter category, while travel times and distances between patient’s location and service points belong to the latter—which is therefore often referred to as spatial accessibility [16]. This distinction is particularly useful when researching EHC accessibility. In emergencies, time is critical and factors pertaining to the geographic distance or travel times are therefore central. Despite the recognition that spatial accessibility is inevitably largely determined by the geographical relationship between patients and service points, much of the existing literature on accessibility to EHC does not account for population’s mobility and the dynamic nature of accessibility [5].

However, increasing availability of population data with high spatiotemporal resolution has opened up possibilities to incorporate temporality in accessibility research. Temporally sensitive accessibility is conceptualized by Järv et al. [4] as depending on three primary factors—people, transport and activities. Accessibility, they argue, depend on where people are located and at what time (people), on the current conditions of the transportations network affecting travel times (transport) and on the destination (activity), referring to factors such as e.g. opening hours or capacity to provide a certain service. Comparing models including one of each of the factors, and all combined, they concluded that the “activity” factors—e.g. the spatial availability of service points and having strict opening hours, had the greatest influence on accessibility measures that include temporal factors, compared to those that do not. Usually, research tends to incorporate one or two of these aspects in models, largely depending on data availability. For example, Rong et al. [17] included live traffic data (transport) to make more realistic measures of equity of spatial accessibility to public medical facilities, while using static population and activity data. In contrast, Tenkanen et al. [18] utilize temporally varying population data (people) to assess variations in accessibility to grocery stores at different times of days.

#### Accessibility analysis in EHC

Accessibility to EHC is commonly calculated by carrying out service area analyses. This is done by first generating travel time buffers from ED’s or ambulance stations, and then summarizing the proportions of the population that fall within these buffers. For example, Lilley et al. [19] summarized the population located within different time thresholds based on travel times between ED’s and the centroids, i.e. geometrical central points of population blocks, in New Zeeland. Similarly, Branas et al. [20] calculated the percentage of the US population that could reach a trauma center using ground ambulances or ambulance helicopters within 45 and 60 min, and Klein et al. [21] calculated the proportion of the population that could reach a burn care facility within 1 or 2 h. Spatial accessibility can also be calculated by finding the fastest route between population block centroids and the closest ED or ambulance station, which can be useful to make statistical comparisons of accessibility across e.g. urban and rural areas (see e.g. [22]).

Incorporating temporality in spatial analysis has increasingly become a focus of research in recent years. This trend has been driven largely by the widespread and almost ubiquitous use of cell phones in many societies, and increasingly available data of population’s location with high spatiotemporal resolution. Such data is useful in many different research fields [23]. For example, during the Covid-19 pandemic, movement data from the mobile network was used to indicate spatial patterns of infection and spreading (see e.g. [24, 25]) and to assess how population mobility changed following implementation of restrictions [26]. In transportation research, mobile phone data has been utilized to study mobility patterns to expand knowledge on where, when and why populations travel [27] and to estimate spatiotemporal variations in accessibility to public transport [28]. A comprehensive summary of the potential applications of location data from the mobile network in research is provided by Blondel et al. [29].

For emergency situations, including temporality in accessibility analysis is of particular interest because the geographical relationship between the closest ED and the patient may impact the chances of survival [1, 13]. Studies that utilize temporally dynamic population data show different findings in terms of where and when population accessibility changes compared to static population data. For example, variations in accessibility over the day has been shown to be greater in areas with high population flows [3]. Comparing the accessibility of rural and urban ED’s when using census tract data and location data from the mobile network, Yun et al. [30] found that rural ED’s were less accessible than urban ED’s when based on static census tract population data. However, they argue, if potential demand is taken into account, the opposite was true. Basing the accessibility analysis on location data from the mobile network, rural ED’s were found to be more accessible than urban ED’s because the population is reduced in the day when people commute into urban areas to work. As a result, the potential number of patients, i.e. demand, is reduced in rural areas, while increasing in urban areas.

### Study area

The study area included all of Sweden, with a population of nearly 10.5 million [31]. Sweden is the third largest country in the European Union in terms of area, whilst the population size is the eleventh largest [32]. There is a north–south divide in terms of population density, where the northern parts of Sweden are generally less populated. In the north, populations are concentrated along the coast where the larger cities are located. This heterogeneity in geographical and demographical conditions, and the fact that the number of ED’s have been reduced from 115 to today’s 68 since 1970 [8], makes Sweden an interesting case study.

**Fig. 1:**
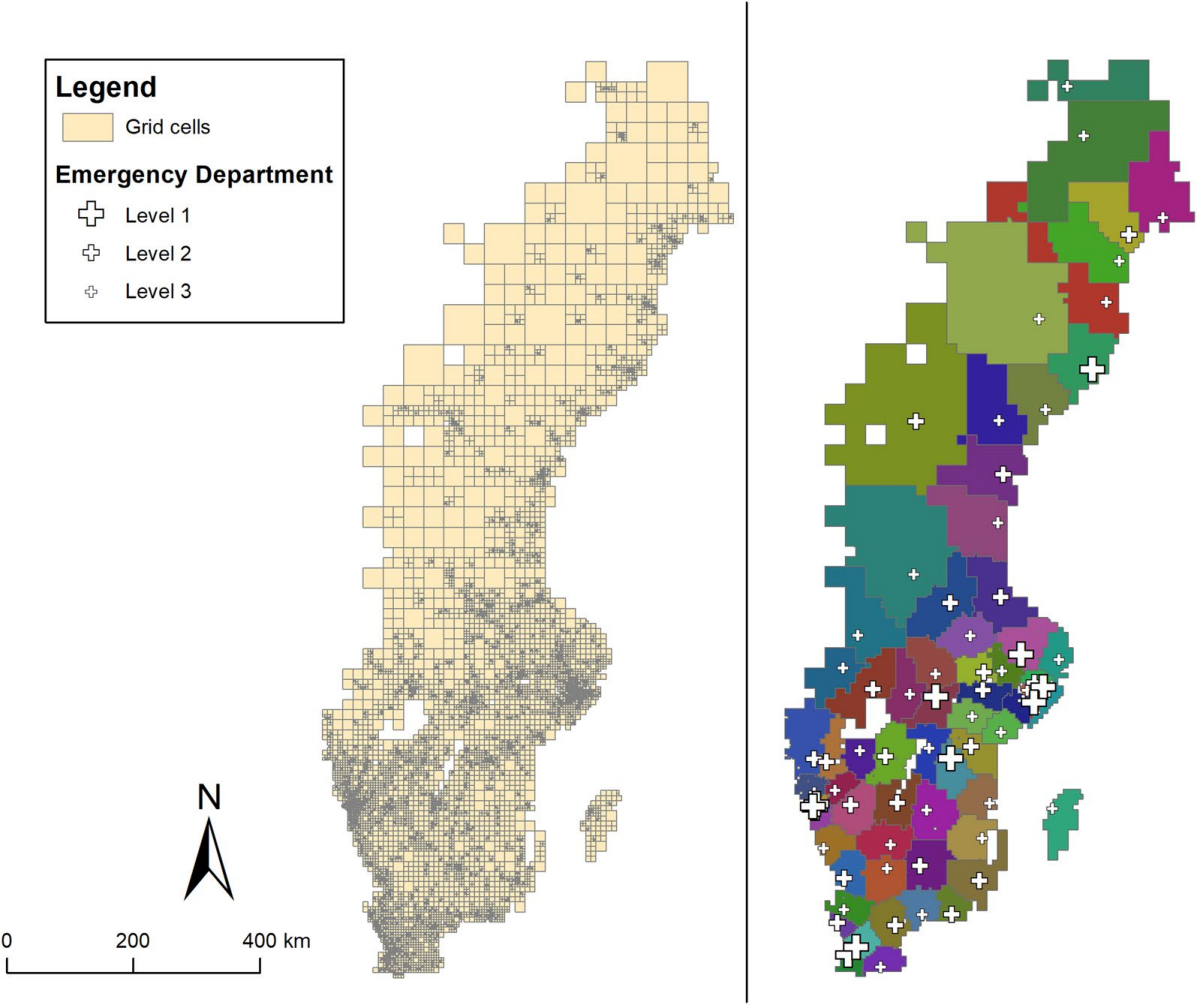
The study area, separated by grid cells used in the analysis (left) and ED catchment areas (right)

In Fig. 1, Sweden is depicted by grid cells (left) and by ED catchment areas (right)—the units of analysis in this study. The varying size of the grid cells reflect varying population density, where smaller grid cells are located in more densely populated areas. Due to the lack of a homogenous definition of what an ED is, it is defined here, derived from an official Swedish report, as a hospital department that can treat patients in acute need of care without the patient having a booked appointment, and also has two or more medical specialist competences [8]. The hospitals in which the ED’s are located are often categorized based on their size and capacities. Although there are no exact definitions of what differentiates them, “Level 1” hospitals are located in the largest cities, have more resources and can treat uncommon conditions while Level 2 and Level 3 hospitals serve smaller populations, have less resources and are often located in smaller cities or rural areas [8].

## Methods

### Data descriptions

Several data types, from different sources, were employed to carry out the analysis. These are presented in Table 1 below. Two separate datasets containing population data were employed. One to represent dynamically moving populations, provided by Telia, Sweden’s largest telecommunications company with mixed public–private ownership, and one containing static population numbers, provided by Statistics Sweden. The former was based on location data from the mobile network, aggregated to a grid. The two grids had overlapping borders which made it possible to combine them in order to compare dynamic and static population counts. However, the grid with static population data from Statistics Sweden had a homogenous size on all grid cells—1 km^2^—while the grid cells in the Telia grid had varying sizes depending on population density and a need to ensure than enough individuals (a minimum of 5) were located within each cell at a certain hour to prevent the possibility of identifying individuals. These grid cells varied in size, ranging between 4096 and 0.25 km^2^.

**Table 1:** Descriptive information about the employed datasets

Dataset	Source	Description	Type	Year
Population data	Telia	Population data based on mobile phone locations compiled on an hour-to-hour basis, aggregated to grid cells	Polygon (point)	2019–2020
	Statistics Sweden	Population data based on place of residence aggregated to Statistics Sweden’s grid cells	Polygon (point)	2019
Road network	The Swedish Transport Administration	Sweden's national road network database, containing all official roads in Sweden	Polyline	2018
Emergency Departments	Manually extracted	Active emergency departments located in hospitals containing two or more specialist competences and the type of hospital	Point	2022
Urban–rural categorization	Swedish Association of Local Authorities and Regions (SALAR)	A system of classifying municipalities as containing; large cities; towns or municipalities close to large cities and rural	–	2017

The dynamic population data consisted of population counts aggregated to 22,763 individual grid cells in a grid covering all of Sweden. The population counts were aggregated based on the number of mobile phones located in a grid cell on an hourly basis for every day between January 1, 2019 and April 1, 2020. In other words, once per hour, the number of phones present in each grid cell were summarized. In total, the dataset contained 229,740,975 observations. Furthermore, the Telia population data contain no device-specific data, and the aggregation process works to make identification of an individual impossible.

To facilitate analysis of differences between urban and rural areas, urban, densely populated and rural area definitions from the Swedish Association of Local Authorities and Regions (SALAR) were used. It is an official national system of categorizing rural and urban areas in Sweden. The definitions rely on population size and commuting behavior—urban municipalities contain less than 20% of the population living in rural areas and have, together with neighboring municipalities, more than 500,000 residents. Densely populated municipalities have less than 50% of the population living rural areas and at least half of the population commute less than 45 min to cities with more than 50,000 residents. Rural municipalities have more than 50% of the population living in rural areas [33].

The road network covering all of Sweden was downloaded from the Swedish national road network database (Nationella vägdatabasen, NVDB) and consisted of 2,135,134 individual road segments, including speed limits for each segment. The road network was split at intersections to assure that each line was connected at an endpoint. This generated an additional 710,761 road segments, as several roads were split into shorter segments. Walking roads, i.e. roads with speed limits below 10 km/h, were removed, and the resulting road network contained 2,541,347 individual segments. Travel times were calculated for each road segment by dividing the length of each segment with the speed limits. All hospital based ED’s in Sweden (N = 68) were extracted manually as.kmz-files from Google Earth based on a list from a recent official report on the current state of Sweden’s EHC system [8], and were then geocoded into a GIS.

### Methodology

To facilitate an analysis where the static and dynamic population sizes could be compared, the first step of the analysis was to combine the grids from Statistic Sweden and from Telia. The two grids were imported to ESRI ArcGIS. Then, the static population data in the grid from Statistics Sweden was incorporated into the Telia grid. While the grids shared borders, the Telia grid cells had varying sizes. In the most densely populated areas, they were smaller than the grid cells from Statistics Sweden. Some had an area of 0.25 km^2^, compared to the grid from Statistics Sweden which had an area of 1 km^2^. Where this occurred, the grid cells from Statistic Sweden were divided into 4 smaller grid cells, each overlapping with the Telia grid cells. The population size was split equally between them, i.e. it was assumed that the static population was equally spread within the grid cell. In less densely populated areas, the Telia grids were larger—up to 4096 km^2^. Where this occurred, the static population size from all the overlapping grid cells in the Statistic Sweden grid was summarized and incorporated as a separate field in the Telia grid. This process generated one grid containing both static and dynamic population data.

The grid and the road network were imported to the GIS in order to calculate the travel times to the closest ED from each zone. All centroids located within 5000 m of the road network were snapped to the closest road network junction. The centroids of 466 grid cells, representing 2% of the total number of grid cells, were either inaccessible from the road network, or were located outside of the Swedish borders. These were generally located in e.g. the archipelago or in mountainous regions and would require either boat or helicopter transport. Also, we did not have access other nations (Norway and Finland) road networks, and could therefore not estimate travel times outside of Sweden. Therefore, these 466 grid cells were omitted from the analysis, and 22,297 of the Telia grid cells (98%) were included in the analysis.

The Closest Facility network analysis, a tool in the ArcGIS suite, was utilized to assess spatial accessibility. It calculates the shortest (quickest) route, based on a road network with information of travel times, by finding the combination of roads between two points with the lowest combined travel time. Travel times were estimated between the centroid of each grid cell and the closest ED, generating one individual route for each grid cell. Each generated route contained an identifying number, which was used as a common denominator to join the data on travel times back to the grid cells. As each grid cell was assigned a route which linked it to the closest ED, a separate variable was generated which indicated which catchment area each grid cell was connected to. Thus, the network analysis also generated the ED catchment areas.

The static populations were summarized in zones located within certain travel time thresholds derived from previous research. To assess how population accessibility vary temporally the dataset was split into different temporal categories—between hours of the day, between weekday and weekends and between months. As the total population sizes varied between the static and dynamic population datasets, we normalized the data before assessing where and when static population data over- or underestimates population size compared to dynamic population data. This was done by comparing the share of the total population that was present within e.g. a certain travel time threshold from an ED, where the share was calculated using the static population data total for the static data, and the dynamic population data total for the dynamic data. To assess whether, and to what degree, static population data over- or underestimates shares compared to dynamic population data, a ratio indicating the relationship between the two datasets was then calculated by dividing the population shares based on static population data with population shares based on dynamic population data. This was done for each hour of the day, separated first by weekdays (Monday-Friday) and weekends (Saturday and Sunday) and then by months.

Then, we compared static and dynamic population datasets for all Swedish ED’s catchment areas at two different time points—at mid-day (13:00) in January and July. This included four steps. First, we visualized over- and underestimations in the ED catchment areas at both time points. Secondly, to complement the spatial patterns observed in the visualizations, Moran’s I tests were conducted to assess whether there were statistically significant clusters in data. Spatial relationships were conceptualized as inversely distanced, meaning neighboring areas have larger influence than those further away. To ensure all ED catchment areas had at least one neighbor, a distance of 137,660 m was automatically set. This conceptualization of spatial relationships was motivated by the fact that the ED catchment areas differ greatly in geographical size, and ensured that neighborhoods did not differ greatly between the smaller ED catchment zones in urban areas (where several EDs are located close to each other) and the larger ED catchment zones in rural areas. Thirdly, analysis of variance (ANOVA) tests were run to see if the clusters of over- and underestimation were also present across different ED types. Fourth, and lastly, as the ANOVA tests indicated statistically significant differences between ED types, we ran Tukey post-hoc tests on the results from the ANOVA tests to assess whether those differences were statistically significant between all types of EDs, or only between some different levels.

### Limitations

A major limitation to this study relates to the dynamic data from the mobile network. Prior to being made available to us it was extrapolated using residential population data. This was done to compensate for the fact that Telia does not control the entire mobile phone market, and thus other operator’s users are not included in the dataset. Extrapolation likely makes the population numbers more realistic, but it entails that the total population size is estimated. The total population present in Sweden also varied over time, which could have several explanations. It could reflect how people move out of, and into, the country. But it is also likely a reflection of limitations to the data itself. For example, only around 30–35% of the population have Telia subscriptions, and some have several phones while others have no phone. Extrapolation made to the data prior to us receiving it therefore, to some degree, induced some uncertainty. Another potential limitation pertains to the Covid-19 pandemic. Travel behaviors may have been affected by recommendations from the public health authorities in Sweden in March, 2020. Due to the short period of time where this may have impacted the data, we did not consider this in the analysis. It is possible that it did impact the results, but likely not to a significant degree.

An official Swedish system of spatial division was employed to delimit urban and rural areas. What is ‘urban’ and what is ‘rural’ is, however, not self-evident—population’s or individuals may identify as urban despite living in a small town that, by official definitions, is considered rural. Urban–rural is thus, perhaps, rather a stratum than two separate, dichotomous categories. Moreover, populations are not necessarily either rural or urban—when rural residents commute to work in larger cities, for example, they would be considered urban during the working hours, and rural when they are at home. Our conceptualization of the urban and rural should be viewed as one possible conceptualization of many alternative ones, and due to the Modifiable Areal Unit Problem (commonly known as MAUP), another division might produce different findings. Another limitation is that the potential impact of weather, traffic and road conditions on travel times were not taken into consideration. However, there are conflicting results about the impact of such factors where studies have shown little effect [4] while others proclaim that they are important to consider [17].

## Results

### Spatial patterns of population accessibility to emergency departments

**Fig. 2:**
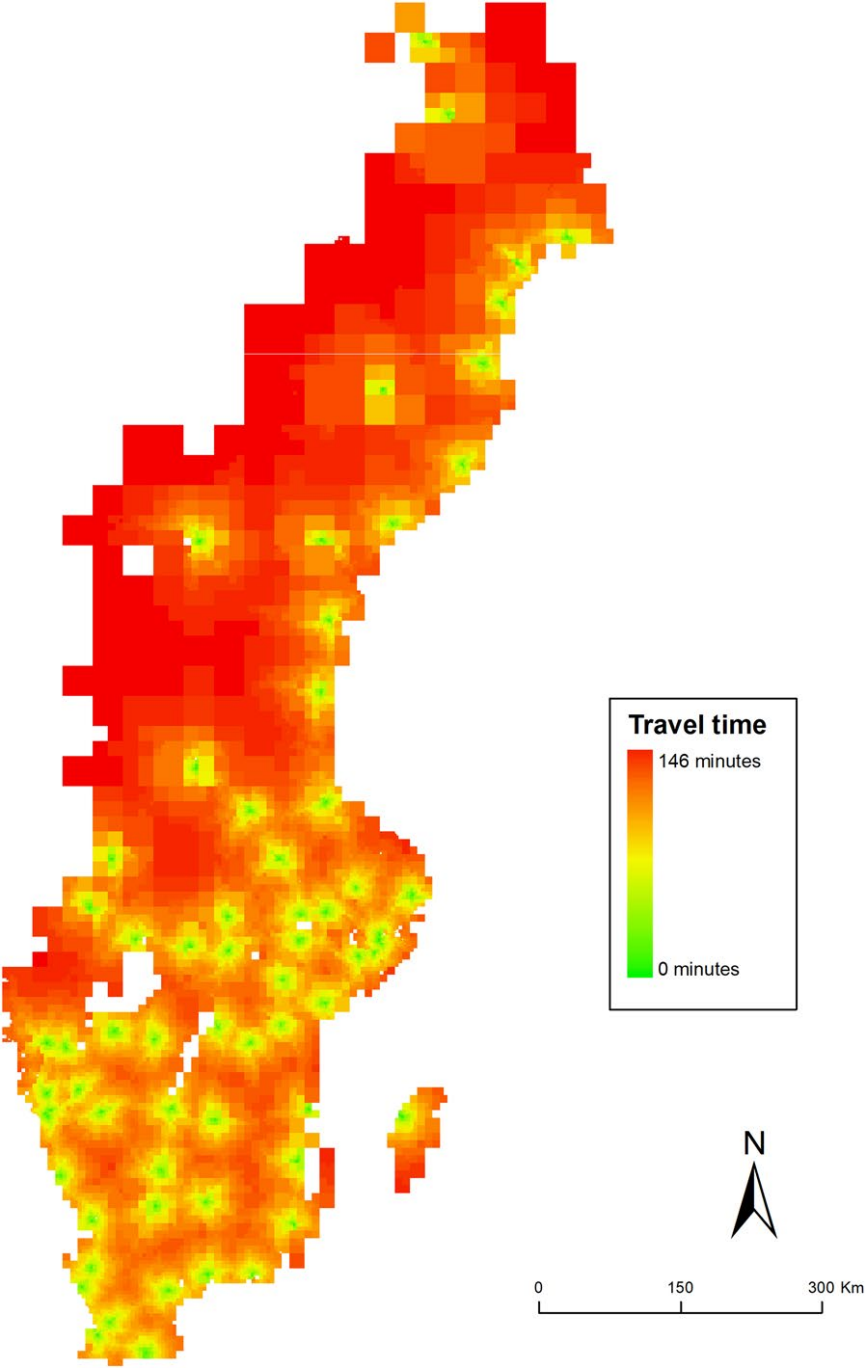
Spatial patterns of accessibility to the closest ED in Sweden

Accessibility was estimated in a network analysis. The spatial patterns of accessibility (see Fig. 2) broadly reflect the population density of Sweden. Along the coasts and urban areas, accessibility is generally high. Further inland, particularly in the northern half of Sweden, large geographic areas have more than one hour to travel to an ED.

Using static population data, we estimated the accessibility for the Swedish population. Table 2 shows descriptive statistics split by urban and rural areas, using the same classifications of accessibility as in Fig. 2. On the national level, there are urban and rural disparities. Nearly 90% of the Swedish population (N = 9,104,076) live within 30 min from an ED and almost half, 48% (N = 4,931,390 individuals) live within 10 min from an ED. In urban areas, more than half of the population (56.3%) live within 10 min of an ED. No urban residents one live further than one hour from an ED. The share of the population that live within 10 min from an ED decreases with rurality—in rural areas, only around a third of the population live within 10 min from an ED. Of the total 132,205 individuals that live further than one hour away from an ED, 124,109 (94%) are rural residents.

**Table 2:** Population accessibility to ED’s separated by travel time intervals and by urban, densely populated and rural areas

Stat	Stat	0–10 min	10–20 min	20–30 min	30–60 min	Above 60 min	Total
Urban	Sum	2,138,989	1,267,713	334,430	61,024	0	3,802,156
	Share	56.26%	33.34%	8.80%	1.60%	–	100.00%
Densely populated	Sum	1,940,608	1,027,009	593,052	352,514	8096	3,921,279
	Share	49.49%	26.19%	15.12%	8.99%	0.21%	100.00%
Rural	Sum	851,793	461,962	488,520	620,963	124,109	2,547,347
	Share	33.44%	18.14%	19.18%	24.38%	4.87%	100.00%
Total	Sum	4,931,390	2,756,684	1,416,002	1,034,501	132,205	10,270,782
	Share	48.01%	26.84%	13.79%	10.07%	1.29%	100%

### Temporal variations of population accessibility—comparing static and dynamic measures

Temporal variations in over- or underestimation of accessibility for the Swedish population were then assessed by estimating a ratio indicating the relationship between static and dynamic population, first between weekdays (Monday–Friday) and weekends (Saturday and Sunday) and then by months. The graphs are interpreted as follows: values above 1 indicate that static population data overestimates the share of the total population compared to dynamic population data, and values below 1 indicate that it underestimates. A value of 0.01 reflects a 1% difference. For full descriptive statistics, see Additional file 1: Table A1–A14.

#### Over- and underestimation on weekends compared to weekdays

**Fig. 3:**
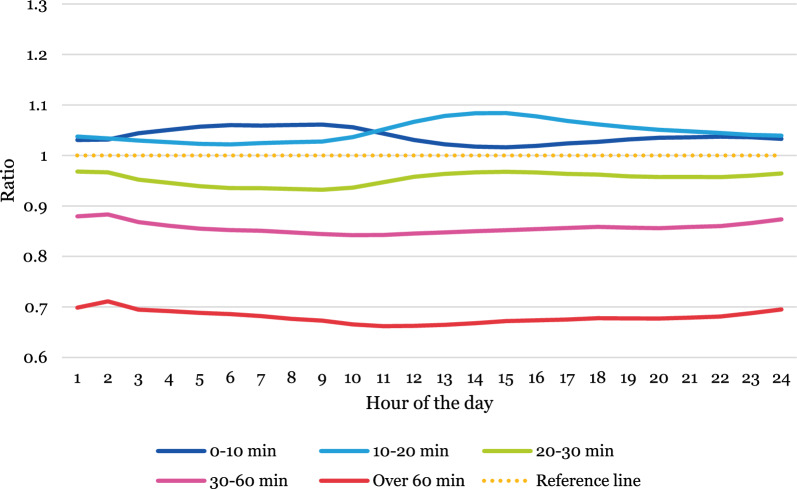
Ratio indicating the difference between population shares located in areas with different accessibility levels estimated from static and dynamic population data in weekends (Saturday and Sunday)

On weekends (Fig. 3), the share of the population located in areas with high accessibility (0–20 min) tend to be slightly overestimated at all times. Areas further away from an ED—with poorer accessibility—have increasingly underestimated shares of the population compared to dynamic population data. In areas located more than 60 min from an ED, static population data underestimates the population by around 30% compared to dynamic population data.

**Fig. 4:**
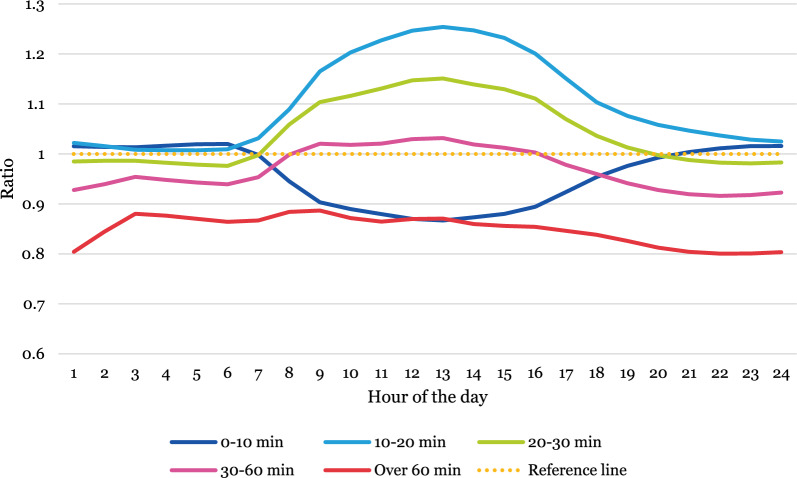
Ratio indicating the difference between population shares located in areas with different accessibility levels estimated from static and dynamic population data in weekdays (Monday–Friday)

In the weekdays (Fig. 4), there is more variation over the course of the day. In the areas with the highest accessibility (0–10 min) the share of the population derived from static population data underestimates by roughly 10% during office hours [8–17] compared to dynamic population data. In areas located between 10 and 60 min from an ED, an opposite trend can be observed where static population data overestimates the share of the total population during office hours—at the most by around 25%. In the most remote areas (more than 60 min from an ED) static population data consistently underestimate the share throughout the day.

#### Over- and underestimation between months

**Fig. 5:**
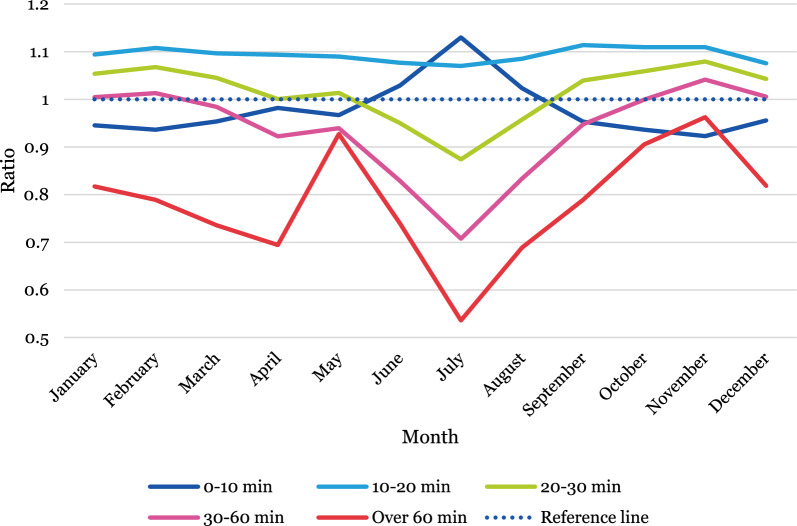
Ratio indicating the difference between population shares located in areas with different accessibility levels estimated from static and dynamic population data, separated by months

In the summer months there is a pattern of increasing underestimation of population shares in areas with poorer accessibility and simultaneous overestimation in the areas with the highest accessibility (see Fig. 5). In areas with more than 60 min to an ED, static population data underestimate shares by around 45% compared to dynamic population data, while overestimating by nearly 15% in areas with less than 10 min travel time to an ED.

Over- and underestimations also vary over the course of the day in different months. Generally, static population data overestimate more in areas with higher accessibility (shorter travel time to an ED) and underestimate in areas with the poorest accessibility (more than one hour travel time to an ED). However, in the areas with the highest accessibility (within 10 min from an ED) population shares are consistently underestimated by up to around 10%, with the exception of July when they are overestimated by up to 17%. Moreover, overestimation in areas with relatively high accessibility (between 10 and 30 min travel time from an ED) appear to increase during the daytime.

### Temporal variation in population sizes per ED catchment areas

**Fig. 6:**
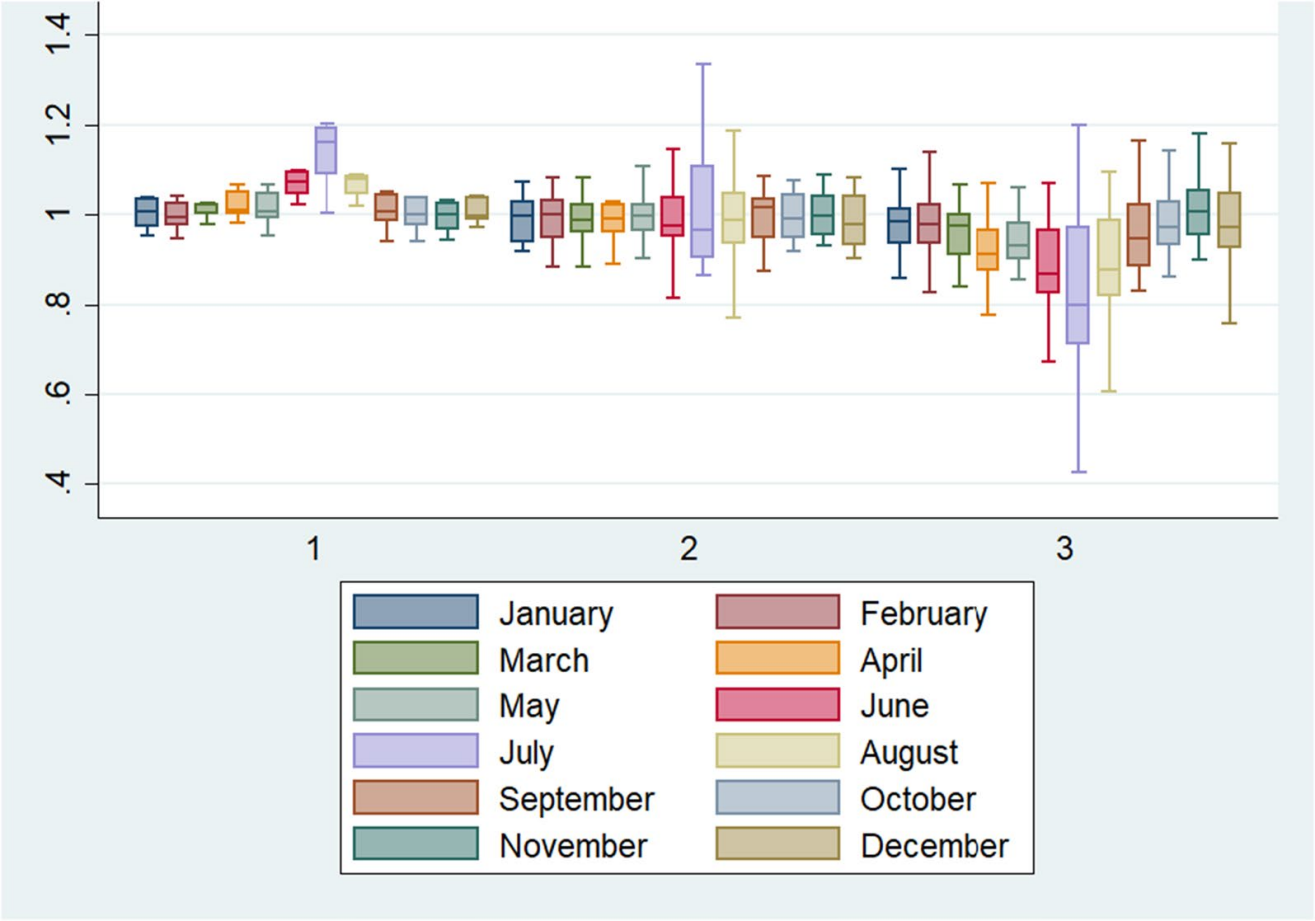
Variation of differences (in percentage) for ED catchment areas by month, separated by ED types

In emergencies, patients are in most cases transported to the closest ED. Larger populations within an ED’s catchment area thus entails increased potential pressure on the ED to provide medical interventions. Population sizes vary as populations, for example, may go on vacation in the summer time, leading to under-or overestimation of population sizes. To illustrate this, we assessed the over- and underestimation of population shares in the ED catchment areas, see Fig. 6. In most months population shares tend to be only slightly over- or underestimated. In the summer months, however, population shares were overestimated in all Level 1 ED’s catchment areas while most Level 3 ED catchment areas had underestimated population shares in the same months.

**Fig. 7:**
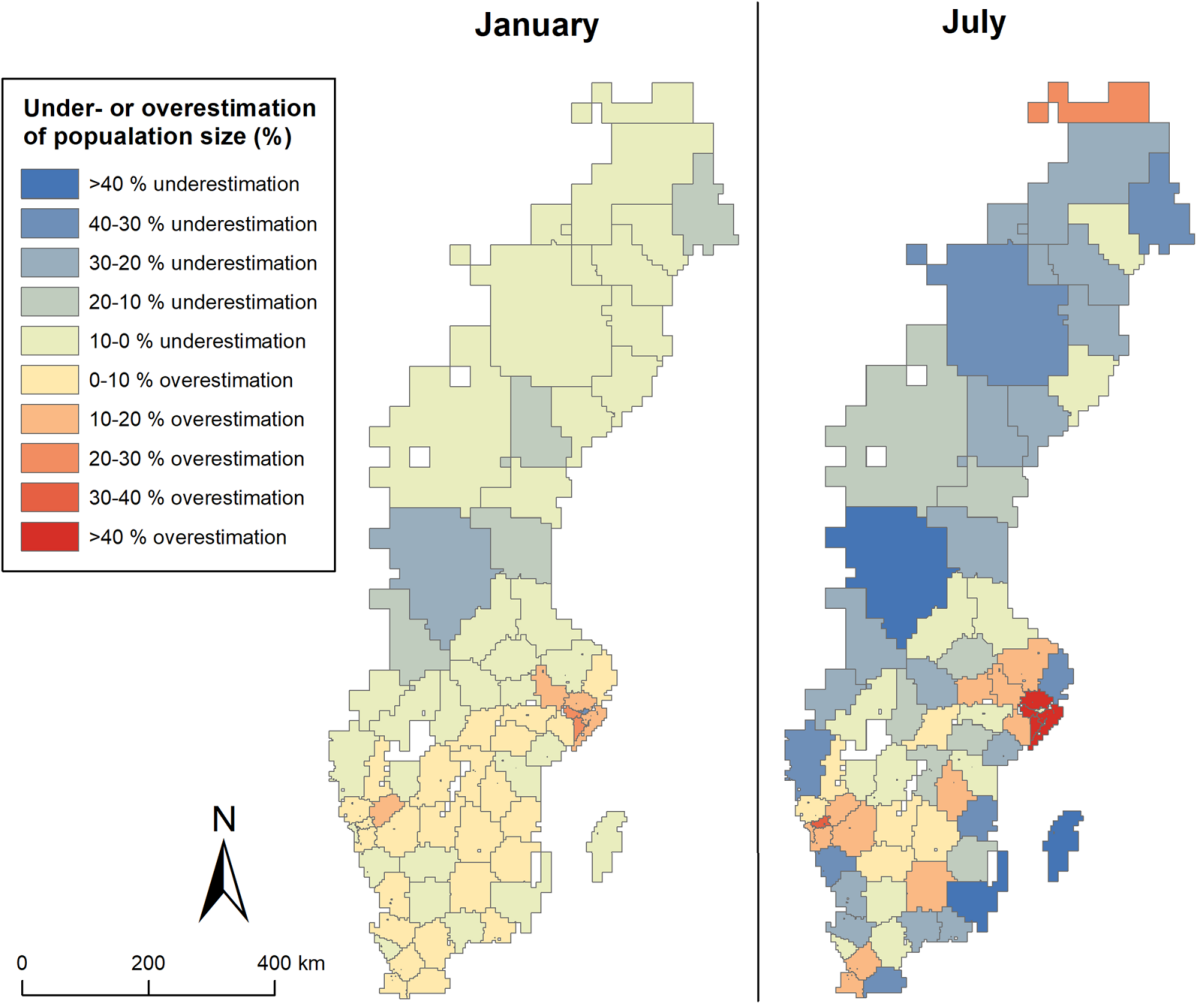
Visualization of over- and underestimation of population sizes in ED catchment areas in Sweden at 13:00 in January (left) and July (right)

Both over- and underestimations were largest in July. To compare how under- and overestimations of population sizes may differ over the seasons, it was compared to January, a month that had less variation and that were similar to most other months of the year. In Fig. 7, the spatial patterns of over- and underestimation of population sizes for all Swedish ED’s catchment areas are visualized at midday (13:00) in January and July. In January, static population data does not appear to over- or underestimate population sizes particularly much. There is a tendency of overestimation in the ED catchment areas in Stockholm, and underestimation in the central parts of Sweden. There is also a slight north–south difference with 0–10% overestimation in southern Sweden, and 0–10% underestimation in northern Sweden. However, the spatial clustering was not statistically significant, indicated by a Moran’s I test (0.01, p = 0.598). In July, however, underestimation is high in coastal areas—the islands Öland and Gotland in the southeast in particular—and in northern Sweden in general. In the largest cities (Stockholm, Gothenburg and Malmö) population sizes are overestimated by more than 30–40% in some ED catchment areas. Unlike January, the Moran’s I test indicted significant clustering in July (0.42, p = 0.000).

A one-way ANOVA test was run to assess whether over- or underestimation differed between ED types. This indicated that there were no significant difference across the three ED levels in January (F(2,65) = 6.08, p = 0.901) but that there was a significant difference in July (F(2,65) = 9.78, p = 0.000). A Tukey post-hoc test revealed that the differences exist between Level 1 and Level 2 ED’s (− 0.211 ± 0.082, p = 0.032) and between Level 1 and Level 3 ED’s (− 0.334 ± 0.077, p = 0.000). On average, in January at 13:00 there was very slight over- or underestimation—less than 2% for each ED type. In July, however, static population data overestimated population sizes by nearly 18% in Level 1 catchment areas while underestimating by nearly 16% in the Level 3 category.

## Discussion

In this paper, we assessed spatiotemporal patterns of population accessibility to ED’s in Sweden. Nearly half of Sweden’s population live within 10 min travel time from an ED, and almost 90% of the population live within 30 min from an ED. As the distribution of ED’s reflect population density, rural areas have lower levels of accessibility than urban. For example, out of the total rural population, nearly 30% live further away than 30 min from an ED, which can be compared to less than 2% of the urban population. On the national level, there is a north–south pattern where accessibility is higher in the south. Again, this reflects the spatial patterns of population density, and the poorer geographic coverage of ED’s in the northern parts of Sweden is not surprising considering the centralization processes that have taken place in Sweden and elsewhere in recent decades.

However, these numbers refer to resident populations. As people go to work or perform other activities, they relocate in space. The “accuracy” of estimates based on resident population data, where it is implicitly assumed that the population is stationary, varies over time. Conventional measures of population accessibility, such as above, are in other words more or less “wrong” at different points in time. For example, in the most central areas with the highest accessibility levels (0–10 min from an ED), population sizes were underestimated—albeit only slightly—during office hours. In areas located 10–60 min from an ED, population sizes were in contrast overestimated by up to 25%. Considering ED’s are located largely based on population density and that centralization of ED’s have focused on allocating resources to cities, this could reflect the fact that large parts of the population commute inwards from surrounding areas during the day. Resident population data is therefore less accurately reflecting real population sizes at this time—if location data from the mobile network is assumed to provide more realistic assumptions of population sizes at different times.

During summer vacation time, around June to September, and specifically in July, overestimation is high also in the most central areas with the highest accessibility level’ (within 10 min from an ED) and there was less variation in the degree of overestimation over the course of the day. This likely reflects the movement of large parts of the urban populations going elsewhere for the vacation, which also explains why there were significant clusters of underestimation of population shares in the catchment areas of smaller, rural ED’s in the July. These are located in areas where many go on vacation, for example in Gotland (an island in southwestern Sweden), along the coasts and in the northern parts of Sweden. These results therefore, in some sense, corroborate previous research by Xia et al. [3], whose results indicated that variations in accessibility over the day is greater in areas with high population flow. However, we also show that there are seasonal variations in how accessibility varies, which also reflect the movement of populations between urban and rural areas.

In relation to planning of EHC resources, our findings show that accessibility analysis based on static, resident population data tends to underestimate population sizes in catchment areas of smaller, rural hospitals. In light of the ongoing centralization of ED’s in Sweden and many other countries, where population sizes represent an important metric indicator in relation to efficiently [34], this is important to recognize because decisions to close a hospital may not be motivated at certain times of the year. During July, a vacation month in Sweden, there were significant clusters of underestimation in places where the resident population tends to be relatively low—in the northern parts of Sweden, and on island and coastal areas. The smaller ED’s located in such places, in other words, serve a larger population than traditional accessibility analyses would suggest at certain times of the year.

The argument here is not that under- or overestimation of population size directly leads to decisions to close an ED or not. However, it is a central metric to guide such decisions. As our findings show that population sizes tend to be underestimated in catchment areas of smaller, rural ED’s—and overestimated in the larger ED’s catchment areas—there is a risk that traditional ways to measure population sizes with static population data contribute knowledge that supports decisions that disfavor rural ED’s. Similar arguments have been made before, for example by Yun et al. [30] whose analysis of accessibility to ED’s showed that using static population data may be erroneous and lead to different conclusions compared to what location data from the mobile network indicates. In extension, as has been noted also by others, urban–rural inequities in access [12] and in health outcomes [35] may be perpetuated and increased. Informing about where and when conventional measures of population accessibility is less accurate in reflecting reality, the use of location data from the mobile network in this study may, as Birkin et al. [7] notes, hopefully contribute to increasing social justice and equality when it comes to allocating EHC resources.

In this study, we found that shares of the population with different levels of accessibility differ between static and dynamic datasets. Moreover, these differences are heterogeneous across space and time. While location data from the mobile network holds a lot of promise for accessibility analyses these differences are important to acknowledge. There are also important problems to be overcome when working with dynamic data that impacted the analysis presented here. First of all, location data from the mobile network is often provided by a specific operator which has a certain share of the population as customers. The entire population, in other words, is not represented in the dataset. To control for that, some extrapolation may have to be done—which was the case with our data—to provide approximations of population sizes at certain times and places. This, of course, has an effect on the data and thus the analysis. One effect is that total population sizes varied greatly between the location data from the mobile network (dynamic) and the resident population data (static), which led us to normalize the data and compare shares instead of absolute population numbers in this analysis.

This study adds to a growing body of research on how temporally sensitive data can be incorporated into accessibility analysis (e.g. [4, 17, 18]). The major contribution of this study is perhaps to show how, when and where conventional static modelling over- or underestimates population accessibility to ED’s. Like Järv et al. [4] points out, this is crucial to facilitate equitable distributions of resources. Mapping out where and when conventional modelling produce uncertain estimations can directly influence the way that resources are distributed. The findings of this study should be of relevance also in other fields of research that utilize population data—e.g. epidemiology [24, 25], mobility [26] and transportation [27, 28]. Location data from the mobile network such as the dataset utilized in this study provides opportunities to make more precise predictions and estimations—both spatial and temporal—of where resources may be needed, which can guide policy making.

### Suggestions for future research

Future research should also compare the temporally varying population sizes in EDs catchment areas to EDs visits, i.e. demand. To what degree does population size present within an ED’s catchment area correlate with demand for services, and how does that correlation vary spatiotemporally? This was not done here because we did not have access to demand data of high enough spatiotemporal resolution. Another interesting direction would be to also assess sociodemographic characteristics of populations, and how it changes over time when population’s move around in space. Are the populations in certain places and times potentially more vulnerable to certain conditions—e.g. medical conditions such as strokes or cardiac arrests—and could such information inform allocation of specializations to certain ED’s? Looking at the capacity at the ED’s to treat patients, and the time that patients need to wait before receiving treatment after having arrived at the ED, would also be of interest. After all, it may not matter how quickly the patient arrives to the ED if he or she cannot acquire adequate care.

Looking further into spatiotemporal variations in accessibility, data such as the mobile network data utilized in this study could facilitate different, and more detailed, aggregations. In the Swedish context it would be particularly interesting to assess how over- or underestimations of population sizes manifest in separate administrative regions, as the EHC system is planned regionally. Further analysis along these lines could also ask broader questions, for example whether public services such as health care provision could be better planned without administrative borders, and what the alternatives could be. Moreover, it would be of interest to make a retrospective study in the Swedish setting to assess whether closures of ED’s in recent decades have been made in places where the population is larger than the static population data indicates. In the future, research should also investigate in greater detail how temporal variations in population sizes can influence planning of ED’s and other EHC resources such as ambulances and specialist competences. Relatedly, dynamic accessibility does not solely depend on the location of people but also on the transportation network and capacity of service. Including capacity, e.g. available hospital beds, and the influence of traffic or public transport on accessibility could shed light on variations in accessibility to Swedish ED’s when controlling for both supply and demand factors—as well as for individuals who may not have a car and needs to travel to the ED in other ways.

## Conclusions

This study takes a step towards utilizing location data from the mobile network in geographical research, and illustrates where, and when, conventional models over- or underestimate population accessibility. Accessibility to ED’s vary spatially in Sweden. Northern Sweden have lower levels of accessibility than southern Sweden, reflecting patterns of population density. Likewise, rural populations tend to have lower levels of accessibility. Resources are, in other words, located in areas where many live. Accessibility also varies temporally, which is not captured by conventional accessibility analysis based on static population data. Population shares tend to be underestimated in the catchment areas of smaller, rural ED’s—especially during summer months, when many are on vacation. Concurrently, population shares tend to be overestimated in urban areas, and in the catchment areas of the larger ED’s.

These results illustrate how planning based on conventional metrics that (implicitly) assume static populations may disfavor rural areas. This information is important because it can help decision makers to allocate resources more efficiently and fairly. Essentially, this article points at how static population counts are always more or less inaccurate reflections of the actual population size at certain places and times. In the light of ongoing centralization of ED’s in Sweden and many other countries, where population sizes often represent an important metric for decision makers when deciding on how to allocate resources, this is important to recognize.

### Supplementary Information

**Additional file 1: Table A1.** Descriptive statistics for weekends. ** Table A2.** Descriptive statistics for weekdays. **Table A3.** Descriptive statistics for January. **Table A4.** Descriptive statistics for February. **Table A5.** Descriptive statistics for March. **Table A6.** Descriptive statistics for April. **Table A7.** Descriptive statistics for May. **Table A8.** Descriptive statistics for June. **Table A9.** Descriptive statistics for July. **Table A10.** Descriptive statistics for August. **Table A11.** Descriptive statistics for September. **Table A12.** Descriptive statistics for October. **Table A13.** Descriptive statistics for November. **Table A14.** Descriptive statistics for December.

## Data Availability

The datasets supporting the conclusions of this article are included within the article (and its additional files).

## Abbreviations

ED: Emergency Department
EHC: Emergency Health Care
GIS: Geographical Information System
ANOVA: Analysis of Variance
