# Supplementary material for: Towards more realistic measures of accessibility to emergency departments in Sweden
Source: Int J Health Geogr. 2024 Mar 2;23:6. doi: 10.1186/s12942-024-00364-9 (PMC10909287; doi:10.1186/s12942-024-00364-9)
Supplement: Supplementary file 1 — Additional file 1: Table A1. Descriptive statistics for weekends. Table A2. Descriptive statistics for weekdays. Table A3. Descriptive statistics for January. Table A4. Descriptive statistics for February. Table A5. Descriptive statistics for March. Table A6. Descriptive statistics for April. Table A7. Descriptive statistics for May. Table A8. Descriptive statistics for June. Table A9. Descriptive statistics for July. Table A10. Descriptive statistics for August. Table A11. Descriptive statistics for September. Table A12. Descriptive statistics for October. Table A13. Descriptive statistics for November. Table A14. Descriptive statistics for December. [file 12942_2024_364_MOESM1_ESM.pdf]

Table A1: Descriptive statistics for weekends.

Time	Static population (shares)					Dynamic population (shares)					Difference (Static - Dynamic)					Ratio (Static/Dynamic)				
	0-10	10-20	20-30	30-60	Over 60	0-10	10-20	20-30	30-60	Over 60	0-10	10-20	20-30	30-60	Over 60	0-10	10-20	20-30	30-60	Over 60
00:00	48,0%	26,8%	13,8%	10,1%	1,3%	46,6%	25,8%	14,3%	11,5%	1,8%	1,4%	1,0%	-0,5%	-1,4%	-0,6%	103,1%	103,8%	96,8%	87,9%	69,8%
01:00	48,0%	26,8%	13,8%	10,1%	1,3%	46,5%	25,9%	14,3%	11,4%	1,8%	1,5%	0,9%	-0,5%	-1,3%	-0,5%	103,1%	103,4%	96,6%	88,3%	71,1%
02:00	48,0%	26,8%	13,8%	10,1%	1,3%	46,0%	26,1%	14,5%	11,6%	1,9%	2,0%	0,8%	-0,7%	-1,5%	-0,6%	104,4%	102,9%	95,2%	86,8%	69,5%
03:00	48,0%	26,8%	13,8%	10,1%	1,3%	45,7%	26,1%	14,6%	11,7%	1,9%	2,3%	0,7%	-0,8%	-1,6%	-0,6%	105,1%	102,6%	94,6%	86,1%	69,2%
04:00	48,0%	26,8%	13,8%	10,1%	1,3%	45,4%	26,2%	14,7%	11,8%	1,9%	2,6%	0,6%	-0,9%	-1,7%	-0,6%	105,7%	102,3%	93,9%	85,5%	68,8%
05:00	48,0%	26,8%	13,8%	10,1%	1,3%	45,3%	26,2%	14,8%	11,8%	1,9%	2,7%	0,6%	-1,0%	-1,7%	-0,6%	106,0%	102,2%	93,5%	85,2%	68,6%
06:00	48,0%	26,8%	13,8%	10,1%	1,3%	45,3%	26,2%	14,8%	11,8%	1,9%	2,7%	0,6%	-1,0%	-1,8%	-0,6%	105,9%	102,5%	93,5%	85,1%	68,2%
07:00	48,0%	26,8%	13,8%	10,1%	1,3%	45,3%	26,1%	14,8%	11,9%	1,9%	2,7%	0,7%	-1,0%	-1,8%	-0,6%	106,0%	102,6%	93,4%	84,8%	67,6%
08:00	48,0%	26,8%	13,8%	10,1%	1,3%	45,2%	26,1%	14,8%	11,9%	1,9%	2,8%	0,7%	-1,0%	-1,9%	-0,6%	106,1%	102,8%	93,2%	84,4%	67,3%
09:00	48,0%	26,8%	13,8%	10,1%	1,3%	45,5%	25,9%	14,7%	12,0%	1,9%	2,5%	0,9%	-0,9%	-1,9%	-0,6%	105,6%	103,6%	93,6%	84,2%	66,5%
10:00	48,0%	26,8%	13,8%	10,1%	1,3%	46,0%	25,5%	14,6%	12,0%	1,9%	2,0%	1,3%	-0,8%	-1,9%	-0,7%	104,3%	105,2%	94,7%	84,3%	66,2%
11:00	48,0%	26,8%	13,8%	10,1%	1,3%	46,6%	25,1%	14,4%	11,9%	1,9%	1,4%	1,7%	-0,6%	-1,8%	-0,7%	103,1%	106,7%	95,8%	84,5%	66,3%
12:00	48,0%	26,8%	13,8%	10,1%	1,3%	47,0%	24,9%	14,3%	11,9%	1,9%	1,0%	1,9%	-0,5%	-1,8%	-0,6%	102,2%	107,8%	96,4%	84,8%	66,5%
13:00	48,0%	26,8%	13,8%	10,1%	1,3%	47,2%	24,7%	14,3%	11,9%	1,9%	0,8%	2,1%	-0,5%	-1,8%	-0,6%	101,8%	108,4%	96,6%	85,0%	66,8%
14:00	48,0%	26,8%	13,8%	10,1%	1,3%	47,2%	24,7%	14,3%	11,8%	1,9%	0,8%	2,1%	-0,5%	-1,8%	-0,6%	101,6%	108,4%	96,8%	85,2%	67,2%
15:00	48,0%	26,8%	13,8%	10,1%	1,3%	47,1%	24,9%	14,3%	11,8%	1,9%	0,9%	1,9%	-0,5%	-1,7%	-0,6%	101,9%	107,7%	96,6%	85,4%	67,4%
16:00	48,0%	26,8%	13,8%	10,1%	1,3%	46,9%	25,1%	14,3%	11,8%	1,9%	1,1%	1,7%	-0,5%	-1,7%	-0,6%	102,4%	106,8%	96,3%	85,6%	67,5%
17:00	48,0%	26,8%	13,8%	10,1%	1,3%	46,7%	25,3%	14,4%	11,7%	1,9%	1,3%	1,6%	-0,5%	-1,7%	-0,6%	102,7%	106,2%	96,2%	85,9%	67,8%
18:00	48,0%	26,8%	13,8%	10,1%	1,3%	46,5%	25,4%	14,4%	11,8%	1,9%	1,5%	1,4%	-0,6%	-1,7%	-0,6%	103,2%	105,6%	95,9%	85,7%	67,7%
19:00	48,0%	26,8%	13,8%	10,1%	1,3%	46,4%	25,5%	14,4%	11,8%	1,9%	1,6%	1,3%	-0,6%	-1,7%	-0,6%	103,5%	105,1%	95,7%	85,6%	67,7%
20:00	48,0%	26,8%	13,8%	10,1%	1,3%	46,3%	25,6%	14,4%	11,7%	1,9%	1,7%	1,2%	-0,6%	-1,7%	-0,6%	103,6%	104,8%	95,8%	85,8%	67,9%
21:00	48,0%	26,8%	13,8%	10,1%	1,3%	46,3%	25,7%	14,4%	11,7%	1,9%	1,7%	1,1%	-0,6%	-1,6%	-0,6%	103,7%	104,4%	95,7%	86,0%	68,1%
22:00	48,0%	26,8%	13,8%	10,1%	1,3%	46,3%	25,8%	14,4%	11,6%	1,9%	1,7%	1,1%	-0,6%	-1,6%	-0,6%	103,6%	104,1%	96,0%	86,6%	68,8%
23:00	48,0%	26,8%	13,8%	10,1%	1,3%	46,5%	25,8%	14,3%	11,5%	1,9%	1,5%	1,0%	-0,5%	-1,5%	-0,6%	103,3%	103,9%	96,4%	87,3%	69,5%

Table A2: Descriptive statistics for weekdays.

Time	Static population (shares)					Dynamic population (shares)					Difference (Static - Dynamic)					Ratio (Static/Dynamic)				
	0-10	10-20	20-30	30-60	Over 60	0-10	10-20	20-30	30-60	Over 60	0-10	10-20	20-30	30-60	Over 60	0-10	10-20	20-30	30-60	Over 60
00:00	48,0%	26,8%	13,8%	10,1%	1,3%	47,3%	26,2%	14,0%	10,9%	1,6%	0,7%	0,6%	-0,2%	-0,8%	-0,3%	101,5%	102,2%	98,5%	92,8%	80,4%
01:00	48,0%	26,8%	13,8%	10,1%	1,3%	47,3%	26,4%	14,0%	10,7%	1,5%	0,7%	0,4%	-0,2%	-0,6%	-0,2%	101,4%	101,6%	98,6%	94,0%	84,4%
02:00	48,0%	26,8%	13,8%	10,1%	1,3%	47,4%	26,6%	14,0%	10,6%	1,5%	0,6%	0,2%	-0,2%	-0,5%	-0,2%	101,3%	100,8%	98,6%	95,4%	88,0%
03:00	48,0%	26,8%	13,8%	10,1%	1,3%	47,2%	26,6%	14,1%	10,6%	1,5%	0,8%	0,2%	-0,2%	-0,6%	-0,2%	101,7%	100,8%	98,2%	94,8%	87,7%
04:00	48,0%	26,8%	13,8%	10,1%	1,3%	47,1%	26,6%	14,1%	10,7%	1,5%	0,9%	0,2%	-0,3%	-0,6%	-0,2%	101,9%	100,7%	97,9%	94,3%	87,0%
05:00	48,0%	26,8%	13,8%	10,1%	1,3%	47,1%	26,6%	14,1%	10,7%	1,5%	0,9%	0,2%	-0,3%	-0,7%	-0,2%	102,0%	100,9%	97,6%	93,9%	86,4%
06:00	48,0%	26,8%	13,8%	10,1%	1,3%	48,1%	26,0%	13,8%	10,6%	1,5%	-0,1%	0,8%	0,0%	-0,5%	-0,2%	99,8%	103,1%	99,8%	95,4%	86,7%
07:00	48,0%	26,8%	13,8%	10,1%	1,3%	50,8%	24,6%	13,0%	10,1%	1,5%	-2,8%	2,2%	0,8%	0,0%	-0,2%	94,5%	108,9%	105,9%	99,8%	88,4%
08:00	48,0%	26,8%	13,8%	10,1%	1,3%	53,1%	23,0%	12,5%	9,9%	1,5%	-5,1%	3,8%	1,3%	0,2%	-0,2%	90,3%	116,5%	110,4%	102,1%	88,7%
09:00	48,0%	26,8%	13,8%	10,1%	1,3%	54,0%	22,3%	12,4%	9,9%	1,5%	-6,0%	4,5%	1,4%	0,2%	-0,2%	89,0%	120,3%	111,6%	101,8%	87,2%
10:00	48,0%	26,8%	13,8%	10,1%	1,3%	54,6%	21,8%	12,2%	9,9%	1,5%	-6,6%	5,0%	1,6%	0,2%	-0,2%	88,0%	122,7%	113,1%	102,1%	86,5%
11:00	48,0%	26,8%	13,8%	10,1%	1,3%	55,2%	21,5%	12,0%	9,8%	1,5%	-7,2%	5,3%	1,8%	0,3%	-0,2%	87,0%	124,7%	114,7%	103,0%	87,0%
12:00	48,0%	26,8%	13,8%	10,1%	1,3%	55,4%	21,4%	12,0%	9,8%	1,5%	-7,4%	5,4%	1,8%	0,3%	-0,2%	86,7%	125,4%	115,1%	103,2%	87,1%
13:00	48,0%	26,8%	13,8%	10,1%	1,3%	55,0%	21,5%	12,1%	9,9%	1,5%	-7,0%	5,3%	1,7%	0,2%	-0,2%	87,3%	124,7%	113,9%	101,9%	86,0%
14:00	48,0%	26,8%	13,8%	10,1%	1,3%	54,5%	21,8%	12,2%	10,0%	1,5%	-6,5%	5,1%	1,6%	0,1%	-0,2%	88,0%	123,2%	112,9%	101,2%	85,6%
15:00	48,0%	26,8%	13,8%	10,1%	1,3%	53,7%	22,3%	12,4%	10,1%	1,5%	-5,7%	4,5%	1,4%	0,0%	-0,2%	89,4%	120,1%	111,1%	100,3%	85,4%
16:00	48,0%	26,8%	13,8%	10,1%	1,3%	52,0%	23,3%	12,9%	10,3%	1,5%	-4,0%	3,5%	0,9%	-0,2%	-0,2%	92,4%	115,1%	107,0%	97,8%	84,6%
17:00	48,0%	26,8%	13,8%	10,1%	1,3%	50,3%	24,3%	13,3%	10,5%	1,5%	-2,3%	2,5%	0,5%	-0,4%	-0,2%	95,4%	110,3%	103,6%	96,0%	83,8%
18:00	48,0%	26,8%	13,8%	10,1%	1,3%	49,2%	24,9%	13,6%	10,7%	1,6%	-1,2%	1,9%	0,2%	-0,6%	-0,3%	97,6%	107,6%	101,3%	94,1%	82,6%
19:00	48,0%	26,8%	13,8%	10,1%	1,3%	48,4%	25,3%	13,8%	10,9%	1,6%	-0,4%	1,5%	0,0%	-0,8%	-0,3%	99,3%	105,8%	99,7%	92,8%	81,3%
20:00	48,0%	26,8%	13,8%	10,1%	1,3%	47,8%	25,6%	14,0%	11,0%	1,6%	0,2%	1,2%	-0,2%	-0,9%	-0,3%	100,4%	104,7%	98,8%	91,9%	80,4%
21:00	48,0%	26,8%	13,8%	10,1%	1,3%	47,5%	25,9%	14,1%	11,0%	1,6%	0,5%	1,0%	-0,2%	-0,9%	-0,3%	101,1%	103,7%	98,3%	91,6%	80,1%
22:00	48,0%	26,8%	13,8%	10,1%	1,3%	47,3%	26,1%	14,1%	11,0%	1,6%	0,7%	0,7%	-0,3%	-0,9%	-0,3%	101,6%	102,9%	98,1%	91,8%	80,1%
23:00	48,0%	26,8%	13,8%	10,1%	1,3%	47,3%	26,2%	14,0%	10,9%	1,6%	0,8%	0,6%	-0,2%	-0,8%	-0,3%	101,6%	102,5%	98,3%	92,3%	80,3%

Table A3: Descriptive statistics for January.

Time	Static population (shares)					Dynamic population (shares)					Difference (Static - Dynamic)					Ratio (Static/Dynamic)				
	0-10	10-20	20-30	30-60	Over 60	0-10	10-20	20-30	30-60	Over 60	0-10	10-20	20-30	30-60	Over 60	0-10	10-20	20-30	30-60	Over 60
00:00	48,0%	26,8%	13,8%	10,1%	1,3%	48,2%	26,2%	13,7%	10,3%	1,6%	-0,2%	0,6%	0,1%	-0,2%	-0,4%	99,6%	102,2%	101,0%	98,1%	78,5%
01:00	48,0%	26,8%	13,8%	10,1%	1,3%	48,0%	26,4%	13,8%	10,3%	1,6%	0,0%	0,4%	0,1%	-0,2%	-0,3%	100,1%	101,4%	100,4%	98,3%	81,2%
02:00	48,0%	26,8%	13,8%	10,1%	1,3%	47,9%	26,5%	13,8%	10,3%	1,6%	0,1%	0,4%	0,0%	-0,2%	-0,3%	100,3%	101,4%	100,1%	97,8%	81,3%
03:00	48,0%	26,8%	13,8%	10,1%	1,3%	47,7%	26,5%	13,9%	10,4%	1,6%	0,3%	0,3%	-0,1%	-0,3%	-0,3%	100,7%	101,2%	99,6%	97,2%	81,2%
04:00	48,0%	26,8%	13,8%	10,1%	1,3%	47,5%	26,5%	13,9%	10,4%	1,6%	0,5%	0,3%	-0,1%	-0,3%	-0,3%	101,0%	101,1%	99,2%	96,7%	80,9%
05:00	48,0%	26,8%	13,8%	10,1%	1,3%	47,5%	26,5%	14,0%	10,5%	1,6%	0,5%	0,3%	-0,1%	-0,4%	-0,3%	101,1%	101,1%	99,0%	96,4%	80,7%
06:00	48,0%	26,8%	13,8%	10,1%	1,3%	48,0%	26,2%	13,8%	10,4%	1,6%	0,0%	0,6%	0,0%	-0,3%	-0,3%	99,9%	102,4%	100,0%	97,1%	81,0%
07:00	48,0%	26,8%	13,8%	10,1%	1,3%	49,5%	25,4%	13,4%	10,1%	1,6%	-1,5%	1,4%	0,4%	-0,1%	-0,3%	97,1%	105,4%	103,1%	99,4%	81,7%
08:00	48,0%	26,8%	13,8%	10,1%	1,3%	50,8%	24,5%	13,1%	10,0%	1,6%	-2,8%	2,3%	0,7%	0,0%	-0,3%	94,5%	109,4%	105,4%	100,5%	81,7%
09:00	48,0%	26,8%	13,8%	10,1%	1,3%	51,3%	24,0%	13,0%	10,0%	1,6%	-3,3%	2,8%	0,8%	0,0%	-0,3%	93,5%	111,7%	106,2%	100,4%	80,6%
10:00	48,0%	26,8%	13,8%	10,1%	1,3%	51,9%	23,6%	12,8%	10,0%	1,6%	-3,9%	3,2%	1,0%	0,1%	-0,3%	92,5%	113,5%	107,5%	100,7%	80,2%
11:00	48,0%	26,8%	13,8%	10,1%	1,3%	52,6%	23,3%	12,6%	9,9%	1,6%	-4,6%	3,6%	1,2%	0,2%	-0,3%	91,3%	115,3%	109,2%	101,7%	80,7%
12:00	48,0%	26,8%	13,8%	10,1%	1,3%	53,0%	23,0%	12,5%	9,8%	1,6%	-5,0%	3,8%	1,3%	0,2%	-0,3%	90,6%	116,4%	110,2%	102,4%	81,1%
13:00	48,0%	26,8%	13,8%	10,1%	1,3%	53,0%	23,0%	12,5%	9,9%	1,6%	-5,0%	3,8%	1,3%	0,2%	-0,3%	90,5%	116,5%	110,3%	102,3%	81,1%
14:00	48,0%	26,8%	13,8%	10,1%	1,3%	52,9%	23,1%	12,5%	9,8%	1,6%	-4,9%	3,7%	1,3%	0,2%	-0,3%	90,7%	116,0%	110,2%	102,4%	81,3%
15:00	48,0%	26,8%	13,8%	10,1%	1,3%	52,4%	23,5%	12,6%	9,9%	1,6%	-4,4%	3,3%	1,2%	0,2%	-0,3%	91,6%	114,2%	109,2%	102,1%	81,3%
16:00	48,0%	26,8%	13,8%	10,1%	1,3%	51,4%	24,1%	12,9%	10,0%	1,6%	-3,4%	2,7%	0,9%	0,1%	-0,3%	93,5%	111,2%	106,8%	100,7%	81,3%
17:00	48,0%	26,8%	13,8%	10,1%	1,3%	50,3%	24,8%	13,2%	10,1%	1,6%	-2,3%	2,0%	0,6%	0,0%	-0,3%	95,4%	108,3%	104,8%	99,6%	81,1%
18:00	48,0%	26,8%	13,8%	10,1%	1,3%	49,5%	25,2%	13,4%	10,3%	1,6%	-1,5%	1,6%	0,4%	-0,2%	-0,3%	97,0%	106,4%	102,9%	98,2%	80,3%
19:00	48,0%	26,8%	13,8%	10,1%	1,3%	48,9%	25,5%	13,6%	10,4%	1,6%	-0,9%	1,3%	0,2%	-0,3%	-0,3%	98,1%	105,1%	101,8%	97,3%	79,5%
20:00	48,0%	26,8%	13,8%	10,1%	1,3%	48,6%	25,7%	13,6%	10,4%	1,6%	-0,6%	1,1%	0,2%	-0,3%	-0,3%	98,8%	104,3%	101,2%	96,9%	79,2%
21:00	48,0%	26,8%	13,8%	10,1%	1,3%	48,3%	25,9%	13,7%	10,4%	1,6%	-0,3%	0,9%	0,1%	-0,4%	-0,3%	99,4%	103,5%	100,7%	96,6%	78,9%
22:00	48,0%	26,8%	13,8%	10,1%	1,3%	48,2%	26,1%	13,7%	10,4%	1,6%	-0,2%	0,8%	0,1%	-0,3%	-0,3%	99,6%	102,9%	100,7%	97,1%	79,0%
23:00	48,0%	26,8%	13,8%	10,1%	1,3%	48,2%	26,1%	13,7%	10,3%	1,6%	-0,2%	0,7%	0,1%	-0,2%	-0,3%	99,5%	102,6%	101,0%	97,7%	79,1%

Table A4: Descriptive statistics for February.

Time	Static population (shares)					Dynamic population (shares)					Difference (Static - Dynamic)					Ratio (Static/Dynamic)				
	0-10	10-20	20-30	30-60	Over 60	0-10	10-20	20-30	30-60	Over 60	0-10	10-20	20-30	30-60	Over 60	0-10	10-20	20-30	30-60	Over 60
00:00	48,0%	26,8%	13,8%	10,1%	1,3%	48,1%	26,0%	13,6%	10,4%	1,8%	-0,1%	0,8%	0,2%	-0,3%	-0,5%	99,8%	103,0%	101,2%	96,8%	70,1%
01:00	48,0%	26,8%	13,8%	10,1%	1,3%	48,2%	26,3%	13,7%	10,2%	1,6%	-0,2%	0,5%	0,1%	-0,2%	-0,3%	99,7%	102,0%	101,0%	98,5%	78,7%
02:00	48,0%	26,8%	13,8%	10,1%	1,3%	48,0%	26,3%	13,7%	10,3%	1,6%	0,0%	0,5%	0,1%	-0,2%	-0,3%	99,9%	102,0%	100,6%	97,9%	79,0%
03:00	48,0%	26,8%	13,8%	10,1%	1,3%	47,8%	26,4%	13,8%	10,4%	1,6%	0,2%	0,5%	0,0%	-0,3%	-0,3%	100,3%	101,7%	100,1%	97,2%	78,9%
04:00	48,0%	26,8%	13,8%	10,1%	1,3%	47,7%	26,4%	13,9%	10,4%	1,6%	0,3%	0,4%	0,0%	-0,3%	-0,3%	100,7%	101,6%	99,7%	96,7%	78,6%
05:00	48,0%	26,8%	13,8%	10,1%	1,3%	47,6%	26,4%	13,9%	10,4%	1,6%	0,4%	0,4%	-0,1%	-0,4%	-0,4%	100,8%	101,6%	99,5%	96,5%	78,4%
06:00	48,0%	26,8%	13,8%	10,1%	1,3%	48,3%	26,0%	13,7%	10,4%	1,6%	-0,2%	0,8%	0,1%	-0,3%	-0,4%	99,5%	103,0%	100,7%	97,3%	78,4%
07:00	48,0%	26,8%	13,8%	10,1%	1,3%	49,8%	25,2%	13,3%	10,1%	1,6%	-1,8%	1,6%	0,6%	0,0%	-0,3%	96,3%	106,5%	104,2%	99,9%	78,9%
08:00	48,0%	26,8%	13,8%	10,1%	1,3%	51,3%	24,2%	12,9%	10,0%	1,6%	-3,3%	2,6%	0,9%	0,1%	-0,3%	93,6%	110,8%	106,8%	101,3%	78,9%
09:00	48,0%	26,8%	13,8%	10,1%	1,3%	51,9%	23,7%	12,8%	10,0%	1,7%	-3,9%	3,2%	1,0%	0,1%	-0,4%	92,5%	113,3%	107,8%	101,3%	77,5%
10:00	48,0%	26,8%	13,8%	10,1%	1,3%	52,6%	23,2%	12,6%	9,9%	1,7%	-4,6%	3,6%	1,2%	0,2%	-0,4%	91,3%	115,5%	109,4%	101,7%	77,0%
11:00	48,0%	26,8%	13,8%	10,1%	1,3%	53,3%	22,8%	12,4%	9,8%	1,7%	-5,3%	4,0%	1,4%	0,3%	-0,4%	90,1%	117,4%	111,3%	102,7%	77,0%
12:00	48,0%	26,8%	13,8%	10,1%	1,3%	53,6%	22,6%	12,3%	9,8%	1,7%	-5,6%	4,2%	1,5%	0,3%	-0,4%	89,5%	118,6%	112,2%	103,3%	76,8%
13:00	48,0%	26,8%	13,8%	10,1%	1,3%	53,6%	22,6%	12,3%	9,8%	1,7%	-5,6%	4,2%	1,5%	0,3%	-0,4%	89,6%	118,6%	112,0%	103,0%	76,1%
14:00	48,0%	26,8%	13,8%	10,1%	1,3%	53,4%	22,7%	12,4%	9,8%	1,7%	-5,4%	4,1%	1,4%	0,3%	-0,4%	89,9%	117,9%	111,6%	102,9%	75,7%
15:00	48,0%	26,8%	13,8%	10,1%	1,3%	52,8%	23,1%	12,5%	9,8%	1,7%	-4,8%	3,7%	1,3%	0,2%	-0,4%	91,0%	115,9%	110,4%	102,4%	74,8%
16:00	48,0%	26,8%	13,8%	10,1%	1,3%	51,6%	23,8%	12,8%	10,0%	1,7%	-3,6%	3,0%	1,0%	0,1%	-0,5%	93,0%	112,6%	107,6%	100,7%	73,8%
17:00	48,0%	26,8%	13,8%	10,1%	1,3%	50,5%	24,5%	13,1%	10,1%	1,8%	-2,5%	2,3%	0,7%	-0,1%	-0,5%	95,0%	109,6%	105,5%	99,5%	73,0%
18:00	48,0%	26,8%	13,8%	10,1%	1,3%	49,7%	24,9%	13,3%	10,3%	1,8%	-1,7%	1,9%	0,5%	-0,2%	-0,5%	96,5%	107,7%	103,8%	98,1%	72,0%
19:00	48,0%	26,8%	13,8%	10,1%	1,3%	49,7%	24,9%	13,3%	10,3%	1,8%	-1,7%	1,9%	0,5%	-0,2%	-0,5%	96,5%	107,7%	103,8%	98,1%	72,0%
20:00	48,0%	26,8%	13,8%	10,1%	1,3%	48,7%	25,4%	13,6%	10,4%	1,8%	-0,7%	1,4%	0,3%	-0,4%	-0,5%	98,5%	105,4%	101,9%	96,5%	70,6%
21:00	48,0%	26,8%	13,8%	10,1%	1,3%	48,4%	25,6%	13,6%	10,5%	1,8%	-0,4%	1,2%	0,2%	-0,4%	-0,5%	99,1%	104,6%	101,4%	96,2%	70,4%
22:00	48,0%	26,8%	13,8%	10,1%	1,3%	48,3%	25,8%	13,6%	10,4%	1,8%	-0,3%	1,0%	0,2%	-0,4%	-0,5%	99,4%	103,9%	101,3%	96,6%	70,4%
23:00	48,0%	26,8%	13,8%	10,1%	1,3%	48,3%	25,8%	13,6%	10,4%	1,8%	-0,3%	1,0%	0,2%	-0,4%	-0,5%	99,4%	103,9%	101,3%	96,6%	70,4%

Table A5: Descriptive statistics for March.

Time	Static population (shares)					Dynamic population (shares)					Difference (Static - Dynamic)					Ratio (Static/Dynamic)				
	0-10	10-20	20-30	30-60	Over 60	0-10	10-20	20-30	30-60	Over 60	0-10	10-20	20-30	30-60	Over 60	0-10	10-20	20-30	30-60	Over 60
00:00	48,0%	26,8%	13,8%	10,1%	1,3%	47,7%	26,2%	13,8%	10,5%	1,8%	0,3%	0,6%	0,1%	-0,4%	-0,5%	100,6%	102,4%	100,4%	95,8%	70,3%
01:00	48,0%	26,8%	13,8%	10,1%	1,3%	47,7%	26,2%	13,8%	10,5%	1,8%	0,3%	0,6%	0,1%	-0,4%	-0,5%	100,6%	102,4%	100,4%	95,8%	70,3%
02:00	48,0%	26,8%	13,8%	10,1%	1,3%	47,5%	26,4%	13,9%	10,5%	1,7%	0,5%	0,5%	-0,1%	-0,4%	-0,5%	101,0%	101,8%	99,5%	96,0%	73,7%
03:00	48,0%	26,8%	13,8%	10,1%	1,3%	47,3%	26,4%	14,0%	10,6%	1,8%	0,7%	0,4%	-0,2%	-0,5%	-0,5%	101,5%	101,6%	98,8%	95,1%	73,6%
04:00	48,0%	26,8%	13,8%	10,1%	1,3%	47,1%	26,4%	14,0%	10,7%	1,8%	0,9%	0,4%	-0,2%	-0,6%	-0,5%	101,9%	101,5%	98,4%	94,5%	73,2%
05:00	48,0%	26,8%	13,8%	10,1%	1,3%	47,1%	26,4%	14,1%	10,7%	1,8%	0,9%	0,4%	-0,3%	-0,6%	-0,5%	102,0%	101,6%	98,2%	94,3%	73,0%
06:00	48,0%	26,8%	13,8%	10,1%	1,3%	47,6%	26,1%	13,9%	10,6%	1,8%	0,4%	0,7%	-0,1%	-0,5%	-0,5%	100,8%	102,8%	99,3%	95,0%	73,3%
07:00	48,0%	26,8%	13,8%	10,1%	1,3%	49,1%	25,3%	13,5%	10,4%	1,7%	-1,1%	1,5%	0,3%	-0,3%	-0,5%	97,8%	105,9%	102,4%	97,4%	73,9%
08:00	48,0%	26,8%	13,8%	10,1%	1,3%	50,3%	24,5%	13,2%	10,2%	1,7%	-2,3%	2,4%	0,6%	-0,2%	-0,5%	95,4%	109,6%	104,5%	98,4%	73,6%
09:00	48,0%	26,8%	13,8%	10,1%	1,3%	50,8%	24,0%	13,1%	10,3%	1,8%	-2,8%	2,8%	0,7%	-0,2%	-0,5%	94,4%	111,7%	105,3%	98,3%	72,5%
10:00	48,0%	26,8%	13,8%	10,1%	1,3%	51,4%	23,7%	13,0%	10,2%	1,8%	-3,4%	3,2%	0,8%	-0,2%	-0,5%	93,5%	113,4%	106,5%	98,5%	72,2%
11:00	48,0%	26,8%	13,8%	10,1%	1,3%	51,9%	23,4%	12,8%	10,2%	1,8%	-3,9%	3,4%	1,0%	-0,1%	-0,5%	92,6%	114,8%	107,7%	99,1%	72,7%
12:00	48,0%	26,8%	13,8%	10,1%	1,3%	52,1%	23,2%	12,8%	10,2%	1,8%	-4,1%	3,6%	1,0%	-0,1%	-0,5%	92,2%	115,4%	108,1%	99,3%	73,0%
13:00	48,0%	26,8%	13,8%	10,1%	1,3%	51,9%	23,3%	12,8%	10,2%	1,8%	-3,9%	3,5%	1,0%	-0,1%	-0,5%	92,5%	115,2%	107,6%	98,8%	72,8%
14:00	48,0%	26,8%	13,8%	10,1%	1,3%	51,7%	23,4%	12,9%	10,2%	1,8%	-3,7%	3,4%	0,9%	-0,1%	-0,5%	92,8%	114,5%	107,3%	98,7%	73,0%
15:00	48,0%	26,8%	13,8%	10,1%	1,3%	51,2%	23,8%	13,0%	10,2%	1,8%	-3,2%	3,0%	0,8%	-0,2%	-0,5%	93,7%	112,8%	106,4%	98,4%	73,0%
16:00	48,0%	26,8%	13,8%	10,1%	1,3%	50,3%	24,3%	13,3%	10,4%	1,8%	-2,3%	2,5%	0,6%	-0,3%	-0,5%	95,5%	110,1%	104,2%	97,3%	72,8%
17:00	48,0%	26,8%	13,8%	10,1%	1,3%	49,4%	24,9%	13,5%	10,5%	1,8%	-1,4%	1,9%	0,3%	-0,4%	-0,5%	97,3%	107,5%	102,5%	96,4%	72,5%
18:00	48,0%	26,8%	13,8%	10,1%	1,3%	48,7%	25,3%	13,7%	10,6%	1,8%	-0,7%	1,5%	0,2%	-0,5%	-0,5%	98,6%	106,0%	101,2%	95,4%	72,0%
19:00	48,0%	26,8%	13,8%	10,1%	1,3%	48,2%	25,6%	13,8%	10,7%	1,8%	-0,2%	1,3%	0,0%	-0,6%	-0,5%	99,6%	104,9%	100,3%	94,6%	71,4%
20:00	48,0%	26,8%	13,8%	10,1%	1,3%	47,9%	25,7%	13,8%	10,7%	1,8%	0,1%	1,1%	0,0%	-0,6%	-0,5%	100,2%	104,2%	99,8%	94,3%	71,0%
21:00	48,0%	26,8%	13,8%	10,1%	1,3%	47,7%	25,9%	13,9%	10,7%	1,8%	0,3%	0,9%	-0,1%	-0,6%	-0,5%	100,6%	103,6%	99,5%	94,2%	70,8%
22:00	48,0%	26,8%	13,8%	10,1%	1,3%	47,6%	26,0%	13,9%	10,7%	1,8%	0,4%	0,8%	-0,1%	-0,6%	-0,5%	100,8%	103,0%	99,6%	94,6%	70,9%
23:00	48,0%	26,8%	13,8%	10,1%	1,3%	47,7%	26,1%	13,8%	10,6%	1,8%	0,3%	0,7%	0,0%	-0,5%	-0,5%	100,7%	102,8%	100,0%	95,1%	70,9%

Table A6: Descriptive statistics for April.

Time	Static population (shares)					Dynamic population (shares)					Difference (Static - Dynamic)					Ratio (Static/Dynamic)				
	0-10	10-20	20-30	30-60	Over 60	0-10	10-20	20-30	30-60	Over 60	0-10	10-20	20-30	30-60	Over 60	0-10	10-20	20-30	30-60	Over 60
00:00	48,0%	26,8%	13,8%	10,1%	1,3%	46,2%	26,1%	14,4%	11,3%	1,9%	1,8%	0,7%	-0,6%	-1,2%	-0,6%	103,8%	102,6%	95,8%	89,1%	68,1%
01:00	48,0%	26,8%	13,8%	10,1%	1,3%	46,2%	26,2%	14,4%	11,3%	1,9%	1,8%	0,6%	-0,6%	-1,2%	-0,6%	103,9%	102,2%	95,9%	89,5%	68,6%
02:00	48,0%	26,8%	13,8%	10,1%	1,3%	46,0%	26,5%	14,5%	11,2%	1,8%	2,0%	0,3%	-0,7%	-1,1%	-0,5%	104,3%	101,3%	95,3%	90,3%	70,2%
03:00	48,0%	26,8%	13,8%	10,1%	1,3%	45,9%	26,5%	14,5%	11,2%	1,8%	2,1%	0,3%	-0,7%	-1,1%	-0,5%	104,6%	101,2%	95,0%	89,8%	70,2%
04:00	48,0%	26,8%	13,8%	10,1%	1,3%	45,7%	26,5%	14,6%	11,3%	1,8%	2,3%	0,3%	-0,8%	-1,2%	-0,6%	105,0%	101,1%	94,5%	89,3%	69,9%
05:00	48,0%	26,8%	13,8%	10,1%	1,3%	45,6%	26,5%	14,7%	11,3%	1,8%	2,4%	0,3%	-0,9%	-1,3%	-0,6%	105,2%	101,1%	94,1%	88,9%	69,7%
06:00	48,0%	26,8%	13,8%	10,1%	1,3%	46,2%	26,2%	14,5%	11,3%	1,8%	1,8%	0,7%	-0,7%	-1,2%	-0,6%	103,8%	102,5%	95,3%	89,6%	70,0%
07:00	48,0%	26,8%	13,8%	10,1%	1,3%	47,7%	25,4%	14,1%	11,0%	1,8%	0,3%	1,4%	-0,3%	-0,9%	-0,5%	100,6%	105,6%	98,2%	91,6%	70,2%
08:00	48,0%	26,8%	13,8%	10,1%	1,3%	48,9%	24,5%	13,8%	10,9%	1,9%	-0,9%	2,3%	0,0%	-0,9%	-0,6%	98,2%	109,4%	100,1%	92,2%	69,4%
09:00	48,0%	26,8%	13,8%	10,1%	1,3%	49,4%	24,0%	13,7%	11,0%	1,9%	-1,4%	2,8%	0,1%	-0,9%	-0,6%	97,2%	111,6%	100,9%	91,8%	67,5%
10:00	48,0%	26,8%	13,8%	10,1%	1,3%	49,9%	23,6%	13,5%	11,0%	1,9%	-1,9%	3,2%	0,3%	-0,9%	-0,7%	96,1%	113,5%	102,2%	91,9%	66,2%
11:00	48,0%	26,8%	13,8%	10,1%	1,3%	50,5%	23,3%	13,4%	10,9%	1,9%	-2,5%	3,5%	0,4%	-0,8%	-0,7%	95,1%	115,1%	103,3%	92,3%	66,1%
12:00	48,0%	26,8%	13,8%	10,1%	1,3%	50,6%	23,2%	13,3%	10,9%	1,9%	-2,6%	3,7%	0,5%	-0,8%	-0,7%	94,8%	115,8%	103,5%	92,3%	66,3%
13:00	48,0%	26,8%	13,8%	10,1%	1,3%	50,4%	23,2%	13,4%	11,0%	1,9%	-2,4%	3,6%	0,4%	-0,9%	-0,7%	95,2%	115,5%	102,8%	91,7%	66,2%
14:00	48,0%	26,8%	13,8%	10,1%	1,3%	50,2%	23,4%	13,5%	11,0%	1,9%	-2,2%	3,5%	0,3%	-0,9%	-0,7%	95,7%	114,8%	102,4%	91,4%	66,3%
15:00	48,0%	26,8%	13,8%	10,1%	1,3%	49,7%	23,7%	13,6%	11,1%	1,9%	-1,7%	3,1%	0,2%	-1,0%	-0,6%	96,6%	113,1%	101,7%	91,1%	66,7%
16:00	48,0%	26,8%	13,8%	10,1%	1,3%	48,8%	24,3%	13,8%	11,2%	1,9%	-0,8%	2,5%	0,0%	-1,1%	-0,6%	98,4%	110,4%	99,8%	90,2%	66,9%
17:00	48,0%	26,8%	13,8%	10,1%	1,3%	47,9%	24,9%	14,1%	11,3%	1,9%	0,1%	1,9%	-0,2%	-1,2%	-0,6%	100,3%	107,7%	98,3%	89,6%	66,9%
18:00	48,0%	26,8%	13,8%	10,1%	1,3%	47,2%	25,3%	14,2%	11,4%	1,9%	0,8%	1,6%	-0,4%	-1,3%	-0,6%	101,7%	106,1%	97,1%	88,7%	66,6%
19:00	48,0%	26,8%	13,8%	10,1%	1,3%	46,7%	25,5%	14,3%	11,5%	1,9%	1,3%	1,3%	-0,5%	-1,4%	-0,7%	102,7%	105,1%	96,4%	88,0%	66,1%
20:00	48,0%	26,8%	13,8%	10,1%	1,3%	46,5%	25,7%	14,4%	11,5%	2,0%	1,5%	1,1%	-0,6%	-1,4%	-0,7%	103,3%	104,3%	95,9%	87,8%	66,0%
21:00	48,0%	26,8%	13,8%	10,1%	1,3%	46,3%	25,9%	14,4%	11,5%	1,9%	1,7%	1,0%	-0,6%	-1,4%	-0,7%	103,7%	103,7%	95,7%	87,9%	66,0%
22:00	48,0%	26,8%	13,8%	10,1%	1,3%	46,2%	26,0%	14,5%	11,5%	1,9%	1,9%	0,8%	-0,6%	-1,4%	-0,6%	104,0%	103,1%	95,5%	88,0%	66,6%
23:00	48,0%	26,8%	13,8%	10,1%	1,3%	46,2%	26,1%	14,4%	11,4%	1,9%	1,8%	0,7%	-0,6%	-1,3%	-0,6%	103,9%	102,8%	95,7%	88,6%	67,4%

Table A7: Descriptive statistics for May.

Time	Static population (shares)					Dynamic population (shares)					Difference (Static - Dynamic)					Ratio (Static/Dynamic)				
	0-10	10-20	20-30	30-60	Over 60	0-10	10-20	20-30	30-60	Over 60	0-10	10-20	20-30	30-60	Over 60	0-10	10-20	20-30	30-60	Over 60
00:00	48,0%	26,8%	13,8%	10,1%	1,3%	47,1%	26,2%	14,3%	11,1%	1,4%	0,9%	0,6%	-0,4%	-1,0%	-0,1%	102,0%	102,4%	96,9%	90,9%	91,6%
01:00	48,0%	26,8%	13,8%	10,1%	1,3%	47,0%	26,3%	14,2%	11,1%	1,4%	1,0%	0,5%	-0,4%	-1,0%	-0,1%	102,0%	102,1%	97,1%	91,2%	92,0%
02:00	48,0%	26,8%	13,8%	10,1%	1,3%	46,7%	26,5%	14,3%	11,0%	1,4%	1,3%	0,3%	-0,5%	-0,9%	-0,1%	102,7%	101,1%	96,3%	91,5%	93,1%
03:00	48,0%	26,8%	13,8%	10,1%	1,3%	46,6%	26,6%	14,4%	11,1%	1,4%	1,4%	0,3%	-0,6%	-1,0%	-0,1%	103,1%	101,0%	95,9%	91,1%	92,7%
04:00	48,0%	26,8%	13,8%	10,1%	1,3%	46,4%	26,6%	14,5%	11,1%	1,4%	1,6%	0,2%	-0,7%	-1,1%	-0,1%	103,5%	100,8%	95,3%	90,5%	92,0%
05:00	48,0%	26,8%	13,8%	10,1%	1,3%	46,3%	26,6%	14,5%	11,2%	1,4%	1,7%	0,2%	-0,7%	-1,1%	-0,1%	103,8%	100,9%	95,0%	90,1%	91,4%
06:00	48,0%	26,8%	13,8%	10,1%	1,3%	46,9%	26,3%	14,4%	11,1%	1,4%	1,1%	0,6%	-0,5%	-1,0%	-0,1%	102,4%	102,2%	96,2%	90,8%	91,5%
07:00	48,0%	26,8%	13,8%	10,1%	1,3%	48,3%	25,5%	13,9%	10,8%	1,4%	-0,3%	1,3%	-0,1%	-0,8%	-0,1%	99,3%	105,2%	99,2%	92,9%	92,3%
08:00	48,0%	26,8%	13,8%	10,1%	1,3%	49,6%	24,6%	13,6%	10,7%	1,4%	-1,6%	2,2%	0,2%	-0,7%	-0,1%	96,7%	109,0%	101,4%	93,9%	92,7%
09:00	48,0%	26,8%	13,8%	10,1%	1,3%	50,2%	24,1%	13,5%	10,8%	1,4%	-2,2%	2,7%	0,3%	-0,7%	-0,1%	95,7%	111,1%	102,0%	93,7%	91,9%
10:00	48,0%	26,8%	13,8%	10,1%	1,3%	50,8%	23,7%	13,4%	10,7%	1,4%	-2,8%	3,1%	0,4%	-0,7%	-0,1%	94,6%	113,0%	103,2%	93,9%	91,9%
11:00	48,0%	26,8%	13,8%	10,1%	1,3%	51,4%	23,4%	13,2%	10,6%	1,4%	-3,4%	3,4%	0,6%	-0,6%	-0,1%	93,5%	114,6%	104,5%	94,7%	92,5%
12:00	48,0%	26,8%	13,8%	10,1%	1,3%	51,6%	23,2%	13,2%	10,6%	1,4%	-3,6%	3,6%	0,6%	-0,5%	-0,1%	93,0%	115,5%	104,9%	94,9%	92,7%
13:00	48,0%	26,8%	13,8%	10,1%	1,3%	51,5%	23,3%	13,2%	10,7%	1,4%	-3,5%	3,6%	0,6%	-0,6%	-0,1%	93,3%	115,3%	104,5%	94,5%	92,4%
14:00	48,0%	26,8%	13,8%	10,1%	1,3%	51,2%	23,4%	13,3%	10,7%	1,4%	-3,2%	3,4%	0,5%	-0,6%	-0,1%	93,7%	114,6%	104,1%	94,2%	92,6%
15:00	48,0%	26,8%	13,8%	10,1%	1,3%	50,7%	23,8%	13,4%	10,7%	1,4%	-2,7%	3,0%	0,4%	-0,7%	-0,1%	94,7%	112,8%	103,2%	93,9%	92,5%
16:00	48,0%	26,8%	13,8%	10,1%	1,3%	49,7%	24,4%	13,6%	10,9%	1,4%	-1,7%	2,4%	0,2%	-0,8%	-0,1%	96,5%	110,0%	101,3%	92,9%	92,2%
17:00	48,0%	26,8%	13,8%	10,1%	1,3%	48,8%	25,0%	13,9%	10,9%	1,4%	-0,8%	1,9%	-0,1%	-0,9%	-0,1%	98,3%	107,4%	99,6%	92,1%	91,8%
18:00	48,0%	26,8%	13,8%	10,1%	1,3%	48,1%	25,3%	14,0%	11,1%	1,4%	-0,1%	1,5%	-0,2%	-1,0%	-0,1%	99,7%	105,8%	98,3%	91,1%	91,1%
19:00	48,0%	26,8%	13,8%	10,1%	1,3%	47,6%	25,6%	14,2%	11,2%	1,4%	0,4%	1,2%	-0,4%	-1,1%	-0,1%	100,8%	104,7%	97,4%	90,3%	90,3%
20:00	48,0%	26,8%	13,8%	10,1%	1,3%	47,3%	25,8%	14,3%	11,2%	1,4%	0,7%	1,0%	-0,4%	-1,1%	-0,1%	101,5%	104,0%	96,9%	89,9%	89,9%
21:00	48,0%	26,8%	13,8%	10,1%	1,3%	47,1%	26,0%	14,3%	11,2%	1,4%	0,9%	0,9%	-0,5%	-1,1%	-0,1%	101,9%	103,3%	96,5%	89,8%	90,0%
22:00	48,0%	26,8%	13,8%	10,1%	1,3%	47,0%	26,1%	14,3%	11,2%	1,4%	1,0%	0,7%	-0,5%	-1,1%	-0,1%	102,2%	102,8%	96,4%	89,9%	90,6%
23:00	48,0%	26,8%	13,8%	10,1%	1,3%	47,0%	26,2%	14,3%	11,2%	1,4%	1,0%	0,7%	-0,5%	-1,1%	-0,1%	102,2%	102,5%	96,6%	90,4%	91,2%

Table A8: Descriptive statistics for June.

Time	Static population (shares)					Dynamic population (shares)					Difference (Static - Dynamic)					Ratio (Static/Dynamic)				
	0-10	10-20	20-30	30-60	Over 60	0-10	10-20	20-30	30-60	Over 60	0-10	10-20	20-30	30-60	Over 60	0-10	10-20	20-30	30-60	Over 60
00:00	48,0%	26,8%	13,8%	10,1%	1,3%	44,8%	26,1%	14,9%	12,4%	1,7%	3,2%	0,7%	-1,1%	-2,3%	-0,5%	107,2%	102,7%	92,4%	81,2%	73,7%
01:00	48,0%	26,8%	13,8%	10,1%	1,3%	44,8%	26,2%	14,9%	12,4%	1,7%	3,2%	0,7%	-1,1%	-2,3%	-0,4%	107,1%	102,5%	92,5%	81,6%	74,2%
02:00	48,0%	26,8%	13,8%	10,1%	1,3%	44,4%	26,4%	15,1%	12,4%	1,7%	3,6%	0,4%	-1,3%	-2,3%	-0,4%	108,2%	101,5%	91,3%	81,6%	74,7%
03:00	48,0%	26,8%	13,8%	10,1%	1,3%	44,2%	26,5%	15,2%	12,4%	1,7%	3,8%	0,4%	-1,4%	-2,3%	-0,4%	108,6%	101,3%	91,0%	81,2%	74,5%
04:00	48,0%	26,8%	13,8%	10,1%	1,3%	44,0%	26,5%	15,3%	12,5%	1,7%	4,0%	0,3%	-1,4%	-2,4%	-0,5%	109,1%	101,1%	90,5%	80,8%	74,0%
05:00	48,0%	26,8%	13,8%	10,1%	1,3%	43,9%	26,5%	15,3%	12,5%	1,7%	4,1%	0,3%	-1,5%	-2,4%	-0,5%	109,3%	101,2%	90,2%	80,6%	73,8%
06:00	48,0%	26,8%	13,8%	10,1%	1,3%	44,6%	26,2%	15,1%	12,4%	1,7%	3,4%	0,6%	-1,3%	-2,3%	-0,5%	107,7%	102,4%	91,4%	81,3%	73,9%
07:00	48,0%	26,8%	13,8%	10,1%	1,3%	45,8%	25,5%	14,7%	12,2%	1,7%	2,2%	1,3%	-0,9%	-2,1%	-0,4%	104,7%	105,1%	93,8%	82,7%	74,3%
08:00	48,0%	26,8%	13,8%	10,1%	1,3%	46,7%	24,9%	14,5%	12,2%	1,7%	1,3%	1,9%	-0,7%	-2,1%	-0,5%	102,9%	107,7%	95,0%	82,9%	74,0%
09:00	48,0%	26,8%	13,8%	10,1%	1,3%	47,0%	24,5%	14,5%	12,2%	1,8%	1,0%	2,3%	-0,7%	-2,1%	-0,5%	102,1%	109,4%	95,5%	82,4%	73,0%
10:00	48,0%	26,8%	13,8%	10,1%	1,3%	47,5%	24,2%	14,3%	12,2%	1,8%	0,5%	2,7%	-0,5%	-2,1%	-0,5%	101,0%	111,0%	96,5%	82,4%	72,6%
11:00	48,0%	26,8%	13,8%	10,1%	1,3%	48,0%	23,8%	14,2%	12,2%	1,8%	0,0%	3,0%	-0,4%	-2,1%	-0,5%	100,0%	112,5%	97,4%	82,6%	72,8%
12:00	48,0%	26,8%	13,8%	10,1%	1,3%	48,1%	23,7%	14,2%	12,2%	1,8%	-0,1%	3,1%	-0,4%	-2,1%	-0,5%	99,7%	113,2%	97,4%	82,5%	72,9%
13:00	48,0%	26,8%	13,8%	10,1%	1,3%	47,9%	23,7%	14,3%	12,3%	1,8%	0,1%	3,1%	-0,5%	-2,2%	-0,5%	100,3%	112,9%	96,7%	81,8%	72,6%
14:00	48,0%	26,8%	13,8%	10,1%	1,3%	47,6%	23,9%	14,4%	12,4%	1,8%	0,4%	2,9%	-0,6%	-2,3%	-0,5%	100,8%	112,2%	96,1%	81,5%	72,7%
15:00	48,0%	26,8%	13,8%	10,1%	1,3%	47,2%	24,2%	14,4%	12,4%	1,8%	0,8%	2,6%	-0,6%	-2,3%	-0,5%	101,7%	110,8%	95,6%	81,4%	73,0%
16:00	48,0%	26,8%	13,8%	10,1%	1,3%	46,5%	24,7%	14,6%	12,4%	1,8%	1,5%	2,2%	-0,8%	-2,4%	-0,5%	103,2%	108,8%	94,4%	81,1%	73,1%
17:00	48,0%	26,8%	13,8%	10,1%	1,3%	45,8%	25,2%	14,8%	12,5%	1,8%	2,2%	1,6%	-1,0%	-2,4%	-0,5%	104,9%	106,5%	93,3%	80,9%	73,1%
18:00	48,0%	26,8%	13,8%	10,1%	1,3%	45,3%	25,5%	14,9%	12,5%	1,8%	2,7%	1,3%	-1,1%	-2,5%	-0,5%	106,1%	105,1%	92,5%	80,4%	72,8%
19:00	48,0%	26,8%	13,8%	10,1%	1,3%	44,9%	25,7%	15,0%	12,6%	1,8%	3,1%	1,1%	-1,2%	-2,5%	-0,5%	107,0%	104,2%	92,0%	80,0%	72,4%
20:00	48,0%	26,8%	13,8%	10,1%	1,3%	44,7%	25,9%	15,0%	12,6%	1,8%	3,3%	1,0%	-1,2%	-2,5%	-0,5%	107,4%	103,7%	91,8%	79,9%	72,3%
21:00	48,0%	26,8%	13,8%	10,1%	1,3%	44,6%	25,9%	15,1%	12,6%	1,8%	3,4%	0,9%	-1,2%	-2,5%	-0,5%	107,6%	103,4%	91,7%	79,9%	72,2%
22:00	48,0%	26,8%	13,8%	10,1%	1,3%	44,6%	26,0%	15,0%	12,6%	1,8%	3,4%	0,8%	-1,2%	-2,5%	-0,5%	107,7%	103,0%	91,8%	80,2%	72,6%
23:00	48,0%	26,8%	13,8%	10,1%	1,3%	44,6%	26,1%	15,0%	12,5%	1,8%	3,4%	0,7%	-1,2%	-2,4%	-0,5%	107,5%	102,7%	92,1%	80,7%	73,2%

Table A9: Descriptive statistics for July.

Time	Static population (shares)					Dynamic population (shares)					Difference (Static - Dynamic)					Ratio (Static/Dynamic)				
	0-10	10-20	20-30	30-60	Over 60	0-10	10-20	20-30	30-60	Over 60	0-10	10-20	20-30	30-60	Over 60	0-10	10-20	20-30	30-60	Over 60
00:00	48,0%	26,8%	13,8%	10,1%	1,3%	41,6%	25,5%	15,9%	14,5%	2,4%	6,4%	1,3%	-2,1%	-4,4%	-1,2%	115,3%	105,1%	86,7%	69,7%	52,6%
01:00	48,0%	26,8%	13,8%	10,1%	1,3%	41,7%	25,6%	15,9%	14,4%	2,4%	6,3%	1,3%	-2,1%	-4,3%	-1,1%	115,0%	104,9%	87,0%	70,1%	53,0%
02:00	48,0%	26,8%	13,8%	10,1%	1,3%	41,4%	25,9%	16,0%	14,2%	2,4%	6,6%	0,9%	-2,2%	-4,2%	-1,1%	115,8%	103,5%	86,1%	70,8%	54,4%
03:00	48,0%	26,8%	13,8%	10,1%	1,3%	41,3%	25,9%	16,1%	14,3%	2,4%	6,7%	0,9%	-2,3%	-4,2%	-1,1%	116,3%	103,4%	85,8%	70,5%	54,4%
04:00	48,0%	26,8%	13,8%	10,1%	1,3%	41,1%	26,0%	16,2%	14,4%	2,4%	6,9%	0,8%	-2,4%	-4,3%	-1,1%	116,9%	103,2%	85,4%	70,1%	54,1%
05:00	48,0%	26,8%	13,8%	10,1%	1,3%	41,0%	26,0%	16,2%	14,4%	2,4%	7,0%	0,8%	-2,4%	-4,3%	-1,1%	117,0%	103,2%	85,1%	70,0%	54,1%
06:00	48,0%	26,8%	13,8%	10,1%	1,3%	41,5%	25,8%	16,1%	14,3%	2,4%	6,5%	1,1%	-2,3%	-4,2%	-1,1%	115,6%	104,1%	85,9%	70,6%	54,3%
07:00	48,0%	26,8%	13,8%	10,1%	1,3%	42,2%	25,3%	15,9%	14,2%	2,4%	5,8%	1,5%	-2,0%	-4,1%	-1,1%	113,7%	105,8%	87,1%	71,0%	54,2%
08:00	48,0%	26,8%	13,8%	10,1%	1,3%	42,5%	25,1%	15,8%	14,2%	2,4%	5,5%	1,8%	-2,0%	-4,2%	-1,1%	113,0%	107,0%	87,4%	70,7%	53,6%
09:00	48,0%	26,8%	13,8%	10,1%	1,3%	42,7%	24,8%	15,8%	14,3%	2,4%	5,3%	2,0%	-2,0%	-4,3%	-1,2%	112,5%	108,1%	87,6%	70,3%	52,8%
10:00	48,0%	26,8%	13,8%	10,1%	1,3%	43,0%	24,5%	15,7%	14,4%	2,5%	5,0%	2,4%	-1,8%	-4,3%	-1,2%	111,7%	109,6%	88,2%	69,9%	52,2%
11:00	48,0%	26,8%	13,8%	10,1%	1,3%	43,4%	24,1%	15,6%	14,5%	2,5%	4,6%	2,7%	-1,7%	-4,4%	-1,2%	110,7%	111,2%	88,8%	69,7%	51,9%
12:00	48,0%	26,8%	13,8%	10,1%	1,3%	43,5%	23,9%	15,6%	14,6%	2,5%	4,5%	2,9%	-1,7%	-4,5%	-1,2%	110,3%	112,2%	88,8%	69,3%	51,7%
13:00	48,0%	26,8%	13,8%	10,1%	1,3%	43,4%	23,8%	15,6%	14,7%	2,5%	4,6%	3,0%	-1,8%	-4,6%	-1,2%	110,6%	112,6%	88,4%	68,8%	51,5%
14:00	48,0%	26,8%	13,8%	10,1%	1,3%	43,3%	23,8%	15,7%	14,7%	2,5%	4,7%	3,0%	-1,9%	-4,6%	-1,2%	111,0%	112,5%	88,0%	68,5%	51,4%
15:00	48,0%	26,8%	13,8%	10,1%	1,3%	43,0%	24,0%	15,7%	14,7%	2,5%	5,0%	2,8%	-1,9%	-4,6%	-1,2%	111,5%	111,7%	87,8%	68,5%	51,4%
16:00	48,0%	26,8%	13,8%	10,1%	1,3%	42,6%	24,3%	15,8%	14,7%	2,5%	5,4%	2,5%	-2,0%	-4,6%	-1,2%	112,6%	110,3%	87,2%	68,6%	51,4%
17:00	48,0%	26,8%	13,8%	10,1%	1,3%	42,1%	24,7%	16,0%	14,7%	2,5%	5,9%	2,1%	-2,2%	-4,6%	-1,2%	113,9%	108,7%	86,5%	68,5%	51,4%
18:00	48,0%	26,8%	13,8%	10,1%	1,3%	41,8%	24,9%	16,0%	14,8%	2,5%	6,2%	1,9%	-2,2%	-4,7%	-1,2%	114,9%	107,7%	86,1%	68,3%	51,3%
19:00	48,0%	26,8%	13,8%	10,1%	1,3%	41,5%	25,1%	16,1%	14,8%	2,5%	6,5%	1,7%	-2,3%	-4,7%	-1,2%	115,5%	107,0%	85,9%	68,1%	51,2%
20:00	48,0%	26,8%	13,8%	10,1%	1,3%	41,4%	25,2%	16,1%	14,8%	2,5%	6,6%	1,6%	-2,3%	-4,7%	-1,2%	115,9%	106,4%	85,8%	68,1%	51,2%
21:00	48,0%	26,8%	13,8%	10,1%	1,3%	41,3%	25,3%	16,1%	14,8%	2,5%	6,7%	1,5%	-2,3%	-4,7%	-1,2%	116,2%	106,0%	85,8%	68,3%	51,3%
22:00	48,0%	26,8%	13,8%	10,1%	1,3%	41,3%	25,4%	16,1%	14,7%	2,5%	6,7%	1,4%	-2,3%	-4,6%	-1,2%	116,3%	105,5%	85,9%	68,4%	51,5%
23:00	48,0%	26,8%	13,8%	10,1%	1,3%	41,4%	25,5%	16,0%	14,6%	2,5%	6,6%	1,3%	-2,2%	-4,5%	-1,2%	115,9%	105,3%	86,2%	69,0%	52,0%

Table A10: Descriptive statistics for August.

Time	Static population (shares)					Dynamic population (shares)					Difference (Static - Dynamic)					Ratio (Static/Dynamic)				
	0-10	10-20	20-30	30-60	Over 60	0-10	10-20	20-30	30-60	Over 60	0-10	10-20	20-30	30-60	Over 60	0-10	10-20	20-30	30-60	Over 60
00:00	48,0%	26,8%	13,8%	10,1%	1,3%	45,2%	25,9%	14,8%	12,3%	1,8%	2,8%	0,9%	-1,0%	-2,2%	-0,5%	106,2%	103,4%	93,4%	82,3%	70,2%
01:00	48,0%	26,8%	13,8%	10,1%	1,3%	45,3%	26,0%	14,7%	12,2%	1,8%	2,7%	0,8%	-0,9%	-2,1%	-0,5%	106,0%	103,1%	93,7%	82,9%	70,8%
02:00	48,0%	26,8%	13,8%	10,1%	1,3%	44,7%	26,3%	14,9%	12,2%	1,8%	3,3%	0,5%	-1,1%	-2,1%	-0,5%	107,3%	102,0%	92,6%	82,5%	70,4%
03:00	48,0%	26,8%	13,8%	10,1%	1,3%	44,6%	26,3%	15,0%	12,3%	1,8%	3,4%	0,5%	-1,2%	-2,2%	-0,6%	107,7%	101,9%	92,1%	81,9%	70,1%
04:00	48,0%	26,8%	13,8%	10,1%	1,3%	44,3%	26,4%	15,1%	12,4%	1,9%	3,7%	0,5%	-1,3%	-2,3%	-0,6%	108,3%	101,8%	91,6%	81,4%	69,4%
05:00	48,0%	26,8%	13,8%	10,1%	1,3%	44,2%	26,3%	15,1%	12,4%	1,9%	3,8%	0,5%	-1,3%	-2,4%	-0,6%	108,6%	101,8%	91,2%	81,0%	68,9%
06:00	48,0%	26,8%	13,8%	10,1%	1,3%	44,8%	26,0%	15,0%	12,3%	1,9%	3,2%	0,8%	-1,2%	-2,2%	-0,6%	107,2%	103,0%	92,2%	81,8%	69,0%
07:00	48,0%	26,8%	13,8%	10,1%	1,3%	46,0%	25,4%	14,6%	12,1%	1,9%	2,0%	1,4%	-0,8%	-2,0%	-0,6%	104,3%	105,6%	94,5%	83,2%	69,4%
08:00	48,0%	26,8%	13,8%	10,1%	1,3%	46,9%	24,7%	14,4%	12,1%	1,9%	1,1%	2,1%	-0,6%	-2,0%	-0,6%	102,3%	108,5%	95,8%	83,4%	68,9%
09:00	48,0%	26,8%	13,8%	10,1%	1,3%	47,2%	24,3%	14,4%	12,2%	1,9%	0,8%	2,5%	-0,6%	-2,1%	-0,6%	101,6%	110,2%	96,2%	82,8%	67,9%
10:00	48,0%	26,8%	13,8%	10,1%	1,3%	47,7%	24,0%	14,2%	12,2%	1,9%	0,3%	2,9%	-0,4%	-2,1%	-0,6%	100,6%	111,9%	97,1%	82,7%	67,6%
11:00	48,0%	26,8%	13,8%	10,1%	1,3%	48,3%	23,6%	14,1%	12,1%	1,9%	-0,3%	3,2%	-0,3%	-2,1%	-0,6%	99,4%	113,5%	98,1%	83,0%	67,9%
12:00	48,0%	26,8%	13,8%	10,1%	1,3%	48,5%	23,4%	14,0%	12,1%	1,9%	-0,5%	3,4%	-0,2%	-2,0%	-0,6%	98,9%	114,5%	98,4%	83,1%	68,0%
13:00	48,0%	26,8%	13,8%	10,1%	1,3%	48,4%	23,4%	14,1%	12,2%	1,9%	-0,4%	3,4%	-0,3%	-2,1%	-0,6%	99,1%	114,6%	98,0%	82,7%	67,8%
14:00	48,0%	26,8%	13,8%	10,1%	1,3%	48,3%	23,5%	14,1%	12,2%	1,9%	-0,3%	3,3%	-0,3%	-2,1%	-0,6%	99,4%	114,2%	97,7%	82,6%	68,0%
15:00	48,0%	26,8%	13,8%	10,1%	1,3%	47,9%	23,8%	14,2%	12,2%	1,9%	0,1%	3,0%	-0,4%	-2,1%	-0,6%	100,2%	112,7%	97,1%	82,5%	68,3%
16:00	48,0%	26,8%	13,8%	10,1%	1,3%	47,1%	24,3%	14,4%	12,3%	1,9%	0,9%	2,5%	-0,6%	-2,2%	-0,6%	102,0%	110,2%	95,7%	82,1%	68,5%
17:00	48,0%	26,8%	13,8%	10,1%	1,3%	46,2%	24,9%	14,7%	12,3%	1,9%	1,8%	1,9%	-0,8%	-2,3%	-0,6%	103,9%	107,7%	94,2%	81,7%	68,4%
18:00	48,0%	26,8%	13,8%	10,1%	1,3%	45,6%	25,2%	14,8%	12,4%	1,9%	2,4%	1,6%	-1,0%	-2,4%	-0,6%	105,2%	106,3%	93,3%	81,1%	68,1%
19:00	48,0%	26,8%	13,8%	10,1%	1,3%	45,3%	25,4%	14,9%	12,5%	1,9%	2,7%	1,4%	-1,1%	-2,4%	-0,6%	106,0%	105,4%	92,9%	80,7%	67,8%
20:00	48,0%	26,8%	13,8%	10,1%	1,3%	45,3%	25,4%	14,9%	12,5%	1,9%	2,7%	1,4%	-1,1%	-2,4%	-0,6%	106,0%	105,4%	92,9%	80,7%	67,8%
21:00	48,0%	26,8%	13,8%	10,1%	1,3%	45,0%	25,7%	14,9%	12,5%	1,9%	3,0%	1,1%	-1,1%	-2,4%	-0,6%	106,7%	104,4%	92,6%	80,6%	67,8%
22:00	48,0%	26,8%	13,8%	10,1%	1,3%	44,9%	25,8%	14,9%	12,5%	1,9%	3,1%	1,0%	-1,1%	-2,4%	-0,6%	106,9%	103,8%	92,6%	80,9%	68,3%
23:00	48,0%	26,8%	13,8%	10,1%	1,3%	45,0%	25,9%	14,9%	12,4%	1,9%	3,0%	0,9%	-1,0%	-2,3%	-0,6%	106,6%	103,5%	93,0%	81,5%	69,1%

Table A11: Descriptive statistics for September.

Time	Static population (shares)					Dynamic population (shares)					Difference (Static - Dynamic)					Ratio (Static/Dynamic)				
	0-10	10-20	20-30	30-60	Over 60	0-10	10-20	20-30	30-60	Over 60	0-10	10-20	20-30	30-60	Over 60	0-10	10-20	20-30	30-60	Over 60
00:00	48,0%	26,8%	13,8%	10,1%	1,3%	47,8%	26,0%	13,9%	10,8%	1,6%	0,2%	0,9%	-0,1%	-0,8%	-0,3%	100,4%	103,3%	99,6%	93,1%	82,8%
01:00	48,0%	26,8%	13,8%	10,1%	1,3%	47,9%	26,0%	13,8%	10,8%	1,5%	0,1%	0,8%	0,0%	-0,7%	-0,3%	100,2%	103,2%	100,0%	93,8%	83,6%
02:00	48,0%	26,8%	13,8%	10,1%	1,3%	47,4%	26,2%	14,0%	10,9%	1,6%	0,6%	0,6%	-0,2%	-0,8%	-0,3%	101,4%	102,3%	98,6%	92,9%	82,0%
03:00	48,0%	26,8%	13,8%	10,1%	1,3%	47,2%	26,3%	14,1%	10,9%	1,6%	0,8%	0,6%	-0,3%	-0,9%	-0,3%	101,8%	102,2%	98,1%	92,2%	81,4%
04:00	48,0%	26,8%	13,8%	10,1%	1,3%	46,9%	26,3%	14,2%	11,0%	1,6%	1,1%	0,5%	-0,4%	-0,9%	-0,3%	102,3%	102,0%	97,5%	91,5%	80,5%
05:00	48,0%	26,8%	13,8%	10,1%	1,3%	46,7%	26,3%	14,2%	11,1%	1,6%	1,3%	0,5%	-0,4%	-1,0%	-0,3%	102,7%	102,0%	97,0%	90,8%	79,1%
06:00	48,0%	26,8%	13,8%	10,1%	1,3%	47,3%	26,0%	14,1%	11,0%	1,6%	0,7%	0,9%	-0,3%	-0,9%	-0,4%	101,5%	103,3%	98,1%	91,4%	78,4%
07:00	48,0%	26,8%	13,8%	10,1%	1,3%	48,9%	25,1%	13,6%	10,7%	1,6%	-0,9%	1,7%	0,2%	-0,7%	-0,3%	98,1%	106,8%	101,6%	93,8%	79,1%
08:00	48,0%	26,8%	13,8%	10,1%	1,3%	50,4%	24,1%	13,3%	10,6%	1,6%	-2,4%	2,7%	0,5%	-0,6%	-0,3%	95,3%	111,4%	103,9%	94,8%	78,9%
09:00	48,0%	26,8%	13,8%	10,1%	1,3%	50,9%	23,6%	13,2%	10,7%	1,7%	-2,9%	3,2%	0,6%	-0,6%	-0,4%	94,3%	113,8%	104,6%	94,3%	77,8%
10:00	48,0%	26,8%	13,8%	10,1%	1,3%	51,4%	23,2%	13,1%	10,7%	1,7%	-3,4%	3,6%	0,8%	-0,6%	-0,4%	93,4%	115,7%	105,8%	94,3%	77,3%
11:00	48,0%	26,8%	13,8%	10,1%	1,3%	52,0%	22,8%	12,9%	10,6%	1,7%	-4,0%	4,0%	0,9%	-0,5%	-0,4%	92,3%	117,4%	107,1%	95,0%	78,0%
12:00	48,0%	26,8%	13,8%	10,1%	1,3%	52,3%	22,7%	12,8%	10,6%	1,6%	-4,3%	4,2%	1,0%	-0,5%	-0,4%	91,8%	118,4%	107,6%	95,3%	78,5%
13:00	48,0%	26,8%	13,8%	10,1%	1,3%	52,1%	22,7%	12,9%	10,6%	1,6%	-4,1%	4,1%	0,9%	-0,5%	-0,4%	92,1%	118,1%	107,1%	95,0%	78,5%
14:00	48,0%	26,8%	13,8%	10,1%	1,3%	52,0%	22,9%	12,9%	10,6%	1,6%	-4,0%	3,9%	0,9%	-0,5%	-0,3%	92,4%	117,2%	106,9%	95,1%	79,2%
15:00	48,0%	26,8%	13,8%	10,1%	1,3%	51,5%	23,3%	13,0%	10,6%	1,6%	-3,5%	3,5%	0,8%	-0,5%	-0,3%	93,2%	115,1%	106,1%	95,1%	80,0%
16:00	48,0%	26,8%	13,8%	10,1%	1,3%	50,4%	24,0%	13,3%	10,7%	1,6%	-2,4%	2,8%	0,5%	-0,6%	-0,3%	95,2%	111,8%	103,7%	94,2%	80,4%
17:00	48,0%	26,8%	13,8%	10,1%	1,3%	49,4%	24,7%	13,5%	10,8%	1,6%	-1,4%	2,1%	0,3%	-0,7%	-0,3%	97,2%	108,7%	102,0%	93,5%	80,6%
18:00	48,0%	26,8%	13,8%	10,1%	1,3%	48,7%	25,1%	13,7%	10,9%	1,6%	-0,7%	1,7%	0,1%	-0,8%	-0,3%	98,6%	106,8%	100,5%	92,5%	80,3%
19:00	48,0%	26,8%	13,8%	10,1%	1,3%	48,2%	25,4%	13,9%	11,0%	1,6%	-0,2%	1,4%	0,0%	-0,9%	-0,3%	99,7%	105,7%	99,7%	91,8%	80,1%
20:00	48,0%	26,8%	13,8%	10,1%	1,3%	47,9%	25,6%	13,9%	11,0%	1,6%	0,1%	1,2%	-0,1%	-0,9%	-0,3%	100,3%	104,9%	99,2%	91,5%	80,0%
21:00	48,0%	26,8%	13,8%	10,1%	1,3%	47,7%	25,7%	14,0%	11,0%	1,6%	0,3%	1,1%	-0,2%	-1,0%	-0,3%	100,7%	104,2%	98,9%	91,3%	80,3%
22:00	48,0%	26,8%	13,8%	10,1%	1,3%	47,6%	25,9%	14,0%	11,0%	1,6%	0,5%	0,9%	-0,2%	-0,9%	-0,3%	101,0%	103,6%	98,8%	91,6%	80,9%
23:00	48,0%	26,8%	13,8%	10,1%	1,3%	47,6%	25,9%	13,9%	10,9%	1,6%	0,4%	0,9%	-0,1%	-0,8%	-0,3%	100,8%	103,4%	99,1%	92,3%	81,8%

Table A12: Descriptive statistics for October.

Time	Static population (shares)					Dynamic population (shares)					Difference (Static - Dynamic)					Ratio (Static/Dynamic)				
	0-10	10-20	20-30	30-60	Over 60	0-10	10-20	20-30	30-60	Over 60	0-10	10-20	20-30	30-60	Over 60	0-10	10-20	20-30	30-60	Over 60
00:00	48,0%	26,8%	13,8%	10,1%	1,3%	48,7%	25,9%	13,6%	10,3%	1,4%	-0,7%	0,9%	0,2%	-0,3%	-0,1%	98,6%	103,4%	101,5%	97,5%	91,2%
01:00	48,0%	26,8%	13,8%	10,1%	1,3%	48,8%	26,0%	13,6%	10,3%	1,4%	-0,8%	0,8%	0,2%	-0,2%	-0,1%	98,4%	103,2%	101,8%	98,1%	92,5%
02:00	48,0%	26,8%	13,8%	10,1%	1,3%	48,5%	26,2%	13,7%	10,3%	1,4%	-0,5%	0,6%	0,2%	-0,2%	-0,1%	99,0%	102,4%	101,1%	98,3%	92,8%
03:00	48,0%	26,8%	13,8%	10,1%	1,3%	48,3%	26,3%	13,7%	10,3%	1,4%	-0,3%	0,5%	0,1%	-0,2%	-0,1%	99,4%	102,0%	100,5%	97,8%	92,8%
04:00	48,0%	26,8%	13,8%	10,1%	1,3%	48,0%	26,3%	13,8%	10,4%	1,4%	0,0%	0,5%	0,0%	-0,3%	-0,1%	99,9%	101,8%	99,9%	97,0%	92,1%
05:00	48,0%	26,8%	13,8%	10,1%	1,3%	47,9%	26,3%	13,9%	10,5%	1,4%	0,1%	0,5%	-0,1%	-0,4%	-0,1%	100,2%	101,8%	99,3%	96,3%	91,2%
06:00	48,0%	26,8%	13,8%	10,1%	1,3%	48,4%	26,0%	13,8%	10,4%	1,4%	-0,4%	0,8%	0,1%	-0,3%	-0,1%	99,2%	103,2%	100,4%	96,7%	90,3%
07:00	48,0%	26,8%	13,8%	10,1%	1,3%	49,9%	25,2%	13,3%	10,2%	1,4%	-1,9%	1,7%	0,5%	-0,1%	-0,1%	96,2%	106,6%	103,6%	98,9%	90,4%
08:00	48,0%	26,8%	13,8%	10,1%	1,3%	51,3%	24,2%	13,0%	10,1%	1,4%	-3,3%	2,6%	0,8%	0,0%	-0,1%	93,6%	110,9%	105,9%	99,9%	90,5%
09:00	48,0%	26,8%	13,8%	10,1%	1,3%	51,8%	23,7%	13,0%	10,1%	1,4%	-3,8%	3,2%	0,8%	0,0%	-0,2%	92,7%	113,3%	106,5%	99,6%	89,4%
10:00	48,0%	26,8%	13,8%	10,1%	1,3%	52,4%	23,2%	12,8%	10,1%	1,5%	-4,4%	3,6%	1,0%	0,0%	-0,2%	91,6%	115,4%	107,8%	99,7%	88,7%
11:00	48,0%	26,8%	13,8%	10,1%	1,3%	53,0%	22,9%	12,6%	10,0%	1,4%	-5,0%	4,0%	1,2%	0,1%	-0,2%	90,5%	117,3%	109,4%	100,5%	89,0%
12:00	48,0%	26,8%	13,8%	10,1%	1,3%	53,4%	22,6%	12,5%	10,0%	1,4%	-5,4%	4,2%	1,3%	0,1%	-0,2%	89,9%	118,5%	110,2%	101,1%	89,5%
13:00	48,0%	26,8%	13,8%	10,1%	1,3%	53,3%	22,6%	12,6%	10,0%	1,4%	-5,3%	4,2%	1,2%	0,1%	-0,2%	90,0%	118,5%	109,8%	100,7%	89,4%
14:00	48,0%	26,8%	13,8%	10,1%	1,3%	53,2%	22,8%	12,6%	10,0%	1,4%	-5,2%	4,0%	1,2%	0,1%	-0,1%	90,3%	117,8%	109,6%	100,7%	90,0%
15:00	48,0%	26,8%	13,8%	10,1%	1,3%	52,6%	23,2%	12,7%	10,0%	1,4%	-4,6%	3,6%	1,1%	0,0%	-0,1%	91,2%	115,7%	108,6%	100,5%	90,6%
16:00	48,0%	26,8%	13,8%	10,1%	1,3%	51,6%	23,9%	13,0%	10,2%	1,4%	-3,6%	3,0%	0,8%	-0,1%	-0,1%	93,1%	112,4%	106,2%	99,2%	90,6%
17:00	48,0%	26,8%	13,8%	10,1%	1,3%	51,6%	23,9%	13,0%	10,2%	1,4%	-3,6%	3,0%	0,8%	-0,1%	-0,1%	93,1%	112,4%	106,2%	99,2%	90,6%
18:00	48,0%	26,8%	13,8%	10,1%	1,3%	49,8%	24,9%	13,5%	10,4%	1,4%	-1,8%	1,9%	0,4%	-0,3%	-0,1%	96,4%	107,5%	102,6%	97,2%	90,1%
19:00	48,0%	26,8%	13,8%	10,1%	1,3%	49,3%	25,3%	13,6%	10,5%	1,4%	-1,2%	1,6%	0,2%	-0,4%	-0,2%	97,5%	106,2%	101,6%	96,3%	89,3%
20:00	48,0%	26,8%	13,8%	10,1%	1,3%	48,9%	25,5%	13,7%	10,5%	1,4%	-0,9%	1,4%	0,1%	-0,4%	-0,2%	98,2%	105,3%	101,0%	95,9%	89,0%
21:00	48,0%	26,8%	13,8%	10,1%	1,3%	48,6%	25,7%	13,7%	10,5%	1,4%	-0,6%	1,1%	0,1%	-0,5%	-0,2%	98,7%	104,4%	100,6%	95,7%	89,1%
22:00	48,0%	26,8%	13,8%	10,1%	1,3%	48,5%	25,8%	13,7%	10,5%	1,4%	-0,5%	1,0%	0,1%	-0,4%	-0,1%	99,0%	103,8%	100,6%	96,0%	89,8%
23:00	48,0%	26,8%	13,8%	10,1%	1,3%	48,6%	25,9%	13,7%	10,4%	1,4%	-0,6%	0,9%	0,1%	-0,3%	-0,1%	98,8%	103,4%	101,0%	96,8%	90,6%

Table A13: Descriptive statistics for November.

Time	Static population (shares)					Dynamic population (shares)					Difference (Static - Dynamic)					Ratio (Static/Dynamic)				
	0-10	10-20	20-30	30-60	Over 60	0-10	10-20	20-30	30-60	Over 60	0-10	10-20	20-30	30-60	Over 60	0-10	10-20	20-30	30-60	Over 60
00:00	48,0%	26,8%	13,8%	10,1%	1,3%	49,4%	26,0%	13,4%	9,9%	1,3%	-1,4%	0,8%	0,5%	0,2%	0,0%	97,2%	103,0%	103,4%	101,8%	96,5%
01:00	48,0%	26,8%	13,8%	10,1%	1,3%	49,2%	26,3%	13,4%	9,8%	1,3%	-1,2%	0,6%	0,4%	0,3%	0,0%	97,6%	102,1%	102,9%	102,8%	97,7%
02:00	48,0%	26,8%	13,8%	10,1%	1,3%	49,0%	26,3%	13,5%	9,9%	1,3%	-1,0%	0,5%	0,3%	0,2%	0,0%	97,9%	102,0%	102,5%	102,0%	97,4%
03:00	48,0%	26,8%	13,8%	10,1%	1,3%	48,8%	26,4%	13,6%	10,0%	1,3%	-0,8%	0,4%	0,2%	0,1%	0,0%	98,4%	101,7%	101,8%	101,1%	96,9%
04:00	48,0%	26,8%	13,8%	10,1%	1,3%	48,6%	26,4%	13,6%	10,0%	1,3%	-0,6%	0,4%	0,2%	0,0%	0,0%	98,9%	101,5%	101,3%	100,4%	96,3%
05:00	48,0%	26,8%	13,8%	10,1%	1,3%	48,5%	26,4%	13,7%	10,1%	1,3%	-0,5%	0,4%	0,1%	0,0%	-0,1%	99,0%	101,5%	100,9%	100,0%	95,7%
06:00	48,0%	26,8%	13,8%	10,1%	1,3%	49,0%	26,1%	13,5%	10,0%	1,4%	-1,0%	0,7%	0,3%	0,1%	-0,1%	98,0%	102,8%	101,9%	100,6%	95,3%
07:00	48,0%	26,8%	13,8%	10,1%	1,3%	50,5%	25,2%	13,1%	9,8%	1,3%	-2,5%	1,6%	0,7%	0,3%	-0,1%	95,0%	106,3%	105,3%	102,8%	95,9%
08:00	48,0%	26,8%	13,8%	10,1%	1,3%	52,0%	24,2%	12,8%	9,7%	1,3%	-4,0%	2,6%	1,0%	0,4%	0,0%	92,3%	110,9%	107,9%	104,1%	96,3%
09:00	48,0%	26,8%	13,8%	10,1%	1,3%	52,6%	23,6%	12,7%	9,7%	1,4%	-4,6%	3,2%	1,1%	0,4%	-0,1%	91,2%	113,5%	108,9%	104,0%	95,3%
10:00	48,0%	26,8%	13,8%	10,1%	1,3%	53,3%	23,2%	12,5%	9,7%	1,4%	-5,3%	3,6%	1,3%	0,4%	-0,1%	90,1%	115,7%	110,4%	104,4%	94,9%
11:00	48,0%	26,8%	13,8%	10,1%	1,3%	54,0%	22,8%	12,3%	9,6%	1,3%	-6,0%	4,0%	1,5%	0,5%	-0,1%	88,9%	117,6%	112,1%	105,5%	95,4%
12:00	48,0%	26,8%	13,8%	10,1%	1,3%	54,4%	22,6%	12,2%	9,5%	1,3%	-6,4%	4,3%	1,6%	0,6%	-0,1%	88,3%	118,9%	113,0%	106,0%	96,2%
13:00	48,0%	26,8%	13,8%	10,1%	1,3%	54,3%	22,6%	12,3%	9,6%	1,3%	-6,3%	4,2%	1,5%	0,5%	-0,1%	88,5%	118,7%	112,6%	105,5%	96,0%
14:00	48,0%	26,8%	13,8%	10,1%	1,3%	54,0%	22,7%	12,3%	9,6%	1,3%	-6,0%	4,1%	1,5%	0,5%	0,0%	88,8%	117,9%	112,2%	105,4%	96,6%
15:00	48,0%	26,8%	13,8%	10,1%	1,3%	53,4%	23,2%	12,4%	9,6%	1,3%	-5,4%	3,6%	1,4%	0,5%	0,0%	89,8%	115,7%	110,9%	105,0%	97,2%
16:00	48,0%	26,8%	13,8%	10,1%	1,3%	52,3%	23,9%	12,8%	9,7%	1,3%	-4,3%	3,0%	1,0%	0,3%	0,0%	91,8%	112,4%	108,2%	103,5%	97,0%
17:00	48,0%	26,8%	13,8%	10,1%	1,3%	51,3%	24,5%	13,0%	9,9%	1,3%	-3,3%	2,3%	0,8%	0,2%	0,0%	93,6%	109,3%	106,2%	102,3%	97,0%
18:00	48,0%	26,8%	13,8%	10,1%	1,3%	50,5%	25,0%	13,2%	10,0%	1,3%	-2,5%	1,8%	0,6%	0,1%	-0,1%	95,1%	107,4%	104,4%	100,9%	96,3%
19:00	48,0%	26,8%	13,8%	10,1%	1,3%	49,9%	25,3%	13,4%	10,1%	1,4%	-1,9%	1,5%	0,4%	0,0%	-0,1%	96,2%	106,0%	103,3%	99,8%	95,3%
20:00	48,0%	26,8%	13,8%	10,1%	1,3%	49,5%	25,5%	13,5%	10,1%	1,4%	-1,5%	1,3%	0,4%	-0,1%	-0,1%	97,0%	105,0%	102,6%	99,4%	94,7%
21:00	48,0%	26,8%	13,8%	10,1%	1,3%	49,2%	25,7%	13,5%	10,2%	1,4%	-1,2%	1,1%	0,3%	-0,1%	-0,1%	97,5%	104,2%	102,3%	99,3%	94,7%
22:00	48,0%	26,8%	13,8%	10,1%	1,3%	49,2%	25,9%	13,5%	10,1%	1,3%	-1,2%	0,9%	0,3%	0,0%	-0,1%	97,6%	103,5%	102,5%	100,0%	95,6%
23:00	48,0%	26,8%	13,8%	10,1%	1,3%	49,3%	26,0%	13,4%	10,0%	1,3%	-1,3%	0,8%	0,4%	0,1%	0,0%	97,4%	103,2%	102,9%	101,0%	96,4%

Table A14: Descriptive statistics for December.

Time	Static population (shares)					Dynamic population (shares)					Difference (Static - Dynamic)					Ratio (Static/Dynamic)				
	0-10	10-20	20-30	30-60	Over 60	0-10	10-20	20-30	30-60	Over 60	0-10	10-20	20-30	30-60	Over 60	0-10	10-20	20-30	30-60	Over 60
00:00	48,0%	26,8%	13,8%	10,1%	1,3%	48,2%	26,2%	13,6%	10,3%	1,8%	-0,2%	0,6%	0,2%	-0,2%	-0,5%	99,6%	102,4%	101,6%	98,3%	73,3%
01:00	48,0%	26,8%	13,8%	10,1%	1,3%	48,3%	26,5%	13,6%	10,0%	1,6%	-0,3%	0,3%	0,2%	0,0%	-0,3%	99,4%	101,3%	101,2%	100,5%	82,6%
02:00	48,0%	26,8%	13,8%	10,1%	1,3%	48,2%	26,5%	13,7%	10,1%	1,6%	-0,2%	0,3%	0,1%	0,0%	-0,3%	99,7%	101,3%	100,9%	99,7%	82,5%
03:00	48,0%	26,8%	13,8%	10,1%	1,3%	47,9%	26,6%	13,8%	10,2%	1,6%	0,1%	0,3%	0,0%	-0,1%	-0,3%	100,2%	101,0%	100,2%	98,8%	82,3%
04:00	48,0%	26,8%	13,8%	10,1%	1,3%	47,7%	26,6%	13,9%	10,3%	1,6%	0,3%	0,2%	0,0%	-0,2%	-0,3%	100,7%	100,8%	99,7%	98,1%	81,8%
05:00	48,0%	26,8%	13,8%	10,1%	1,3%	47,6%	26,6%	13,9%	10,3%	1,6%	0,4%	0,2%	-0,1%	-0,2%	-0,3%	100,8%	100,8%	99,4%	97,8%	81,3%
06:00	48,0%	26,8%	13,8%	10,1%	1,3%	48,1%	26,3%	13,8%	10,3%	1,6%	-0,1%	0,5%	0,0%	-0,2%	-0,3%	99,9%	101,9%	100,2%	98,2%	81,3%
07:00	48,0%	26,8%	13,8%	10,1%	1,3%	49,2%	25,7%	13,5%	10,1%	1,6%	-1,2%	1,1%	0,3%	0,0%	-0,3%	97,6%	104,4%	102,5%	99,8%	81,8%
08:00	48,0%	26,8%	13,8%	10,1%	1,3%	50,2%	24,9%	13,2%	10,0%	1,6%	-2,2%	1,9%	0,6%	0,1%	-0,3%	95,6%	107,6%	104,3%	100,6%	81,9%
09:00	48,0%	26,8%	13,8%	10,1%	1,3%	50,8%	24,4%	13,1%	10,0%	1,6%	-2,8%	2,4%	0,7%	0,1%	-0,3%	94,5%	109,7%	105,4%	100,5%	80,8%
10:00	48,0%	26,8%	13,8%	10,1%	1,3%	51,6%	23,9%	12,9%	10,0%	1,6%	-3,6%	2,9%	0,9%	0,1%	-0,3%	93,0%	112,0%	107,3%	101,2%	80,3%
11:00	48,0%	26,8%	13,8%	10,1%	1,3%	52,5%	23,5%	12,6%	9,9%	1,6%	-4,4%	3,3%	1,2%	0,2%	-0,3%	91,5%	114,2%	109,4%	102,3%	80,7%
12:00	48,0%	26,8%	13,8%	10,1%	1,3%	52,9%	23,2%	12,5%	9,8%	1,6%	-4,9%	3,6%	1,3%	0,3%	-0,3%	90,7%	115,7%	110,7%	103,0%	80,6%
13:00	48,0%	26,8%	13,8%	10,1%	1,3%	53,0%	23,1%	12,5%	9,8%	1,6%	-5,0%	3,7%	1,3%	0,3%	-0,3%	90,6%	115,9%	110,8%	102,9%	80,1%
14:00	48,0%	26,8%	13,8%	10,1%	1,3%	52,8%	23,3%	12,5%	9,8%	1,6%	-4,8%	3,6%	1,3%	0,3%	-0,3%	90,9%	115,3%	110,5%	102,8%	79,6%
15:00	48,0%	26,8%	13,8%	10,1%	1,3%	52,2%	23,6%	12,7%	9,9%	1,6%	-4,2%	3,2%	1,2%	0,2%	-0,3%	92,0%	113,5%	109,1%	101,9%	78,7%
16:00	48,0%	26,8%	13,8%	10,1%	1,3%	51,1%	24,2%	12,9%	10,0%	1,7%	-3,1%	2,6%	0,9%	0,0%	-0,4%	93,9%	110,7%	106,6%	100,3%	77,6%
17:00	48,0%	26,8%	13,8%	10,1%	1,3%	50,1%	24,8%	13,2%	10,2%	1,7%	-2,1%	2,0%	0,6%	-0,1%	-0,4%	95,8%	108,2%	104,4%	98,9%	76,4%
18:00	48,0%	26,8%	13,8%	10,1%	1,3%	49,3%	25,2%	13,4%	10,3%	1,7%	-1,3%	1,6%	0,4%	-0,3%	-0,4%	97,4%	106,4%	102,7%	97,4%	75,2%
19:00	48,0%	26,8%	13,8%	10,1%	1,3%	48,7%	25,5%	13,6%	10,5%	1,7%	-0,7%	1,3%	0,2%	-0,4%	-0,4%	98,7%	105,1%	101,4%	96,3%	74,1%
20:00	48,0%	26,8%	13,8%	10,1%	1,3%	48,3%	25,7%	13,7%	10,5%	1,8%	-0,3%	1,1%	0,1%	-0,4%	-0,5%	99,4%	104,2%	100,8%	95,9%	73,5%
21:00	48,0%	26,8%	13,8%	10,1%	1,3%	48,1%	25,9%	13,7%	10,5%	1,8%	-0,1%	0,9%	0,1%	-0,4%	-0,5%	99,8%	103,5%	100,5%	95,9%	73,2%
22:00	48,0%	26,8%	13,8%	10,1%	1,3%	48,0%	26,1%	13,7%	10,4%	1,8%	0,0%	0,7%	0,1%	-0,4%	-0,5%	99,9%	102,9%	100,7%	96,6%	73,4%
23:00	48,0%	26,8%	13,8%	10,1%	1,3%	48,2%	26,1%	13,6%	10,3%	1,7%	-0,2%	0,7%	0,2%	-0,2%	-0,5%	99,6%	102,7%	101,3%	97,6%	73,8%
